# Artificial intelligence and the analysis of cryo-EM data provide structural insight into the molecular mechanisms underlying LN-lamininopathies

**DOI:** 10.1038/s41598-023-45200-5

**Published:** 2023-10-19

**Authors:** Arkadiusz W. Kulczyk

**Affiliations:** 1 Institute for Quantitative Biomedicine, Rutgers University, 174 Frelinghuysen Road, Piscataway, NJ 08854 USA; 2 Department of Biochemistry & Microbiology, Rutgers University, 75 Lipman Drive, New Brunswick, NJ 08901 USA

**Keywords:** Cryoelectron microscopy, Computational models

## Abstract

Laminins (Lm) are major components of basement membranes (BM), which polymerize to form a planar lattice on cell surface. Genetic alternations of Lm affect their oligomerization patterns and lead to failures in BM assembly manifesting in a group of human disorders collectively defined as Lm N-terminal domain lamininopathies (LN-lamininopathies). We have employed a recently determined cryo-EM structure of the Lm polymer node, the basic repeating unit of the Lm lattice, along with structure prediction and modeling to systematically analyze structures of twenty-three pathogenic Lm polymer nodes implicated in human disease. Our analysis provides the detailed mechanistic explanation how Lm mutations lead to failures in Lm polymerization underlining LN-lamininopathies. We propose the new categorization scheme of LN-lamininopathies based on the insight gained from the structural analysis. Our results can help to facilitate rational drug design aiming in the treatment of Lm deficiencies.

## Introduction

**Figure 1:**
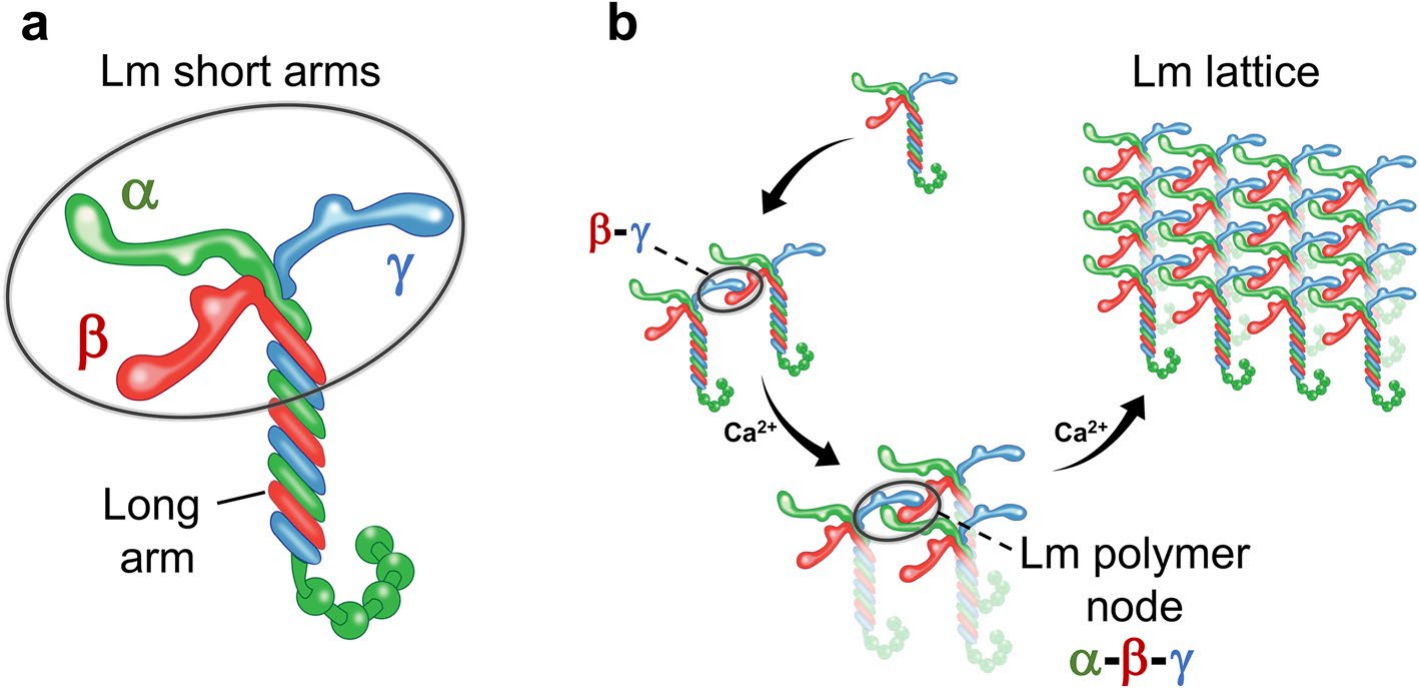
A model for Lm assembly and polymerization. (**a**) The heterotrimeric Lm complex is assembled upon joining of α, β, and γ subunits. Lm short and long arms are labeled in the figure. (**b**) Calcium independent formation of β-γ dimers is followed by a calcium dependent association of α with the dimer, and the extension of Lm lattice. One of the trimeric Lm polymer nodes formed by interacting short arms from α, β and γ is highlighted.

**Figure 2:**
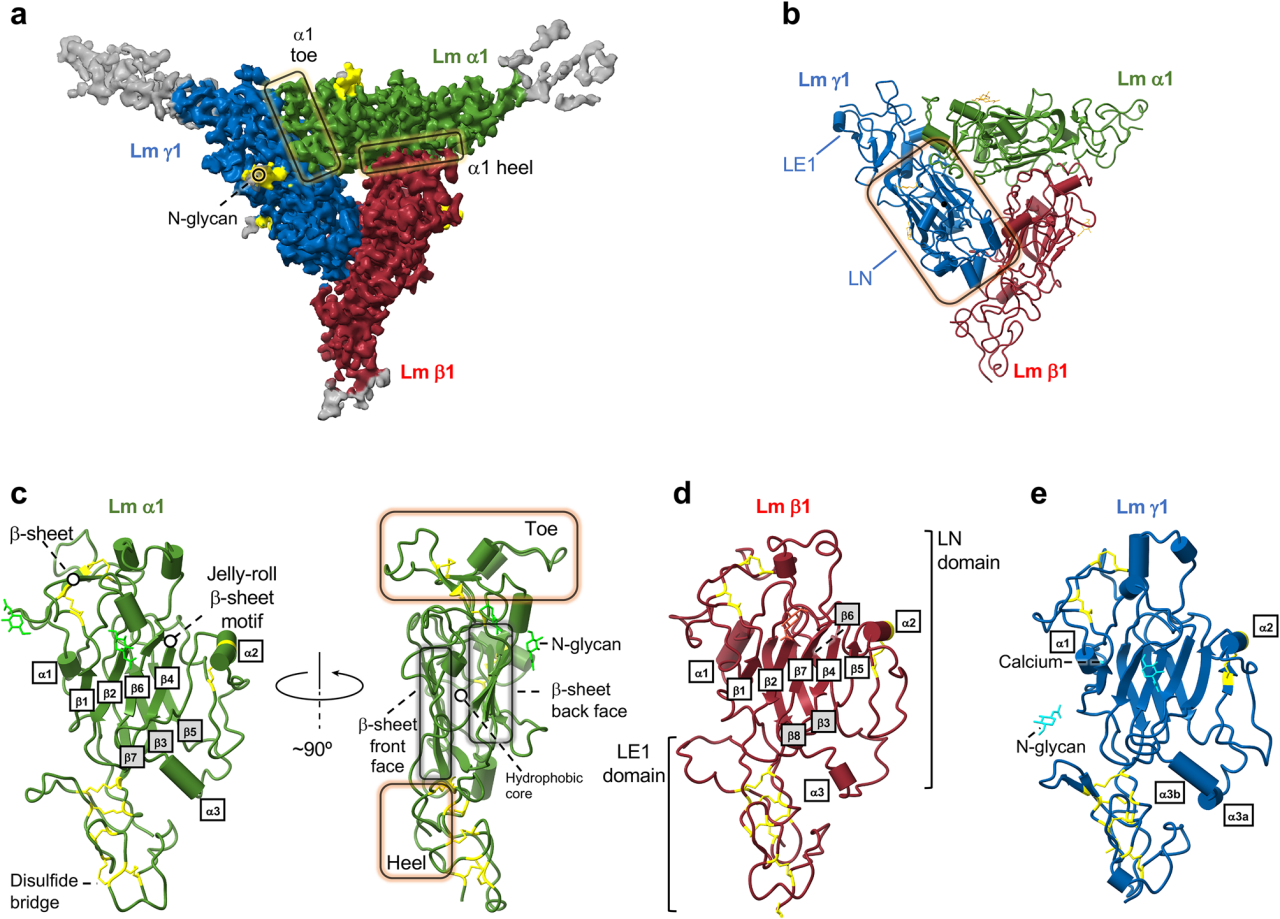
Cryo-EM structure of Lm polymer node. (**a**) The cryo-EM map (EMD-27542^13^) is color-coded. Lm α1, β1 and γ1 are shown in green, red and blue, respectively. Flexible parts of the Coulomb map with no model built are displayed in gray. The N-glycans are colored in yellow. (**b**) A cartoon representation of the Lm α1β1γ1 polymer node’s structure (PDB ID: 8DMK^13^). The LN and LE1 domains are labeled in γ1 subunit. A calcium ion is shown as a black sphere. Atomic models^13^ of (**c**) Lm α1, (**d**) Lm β1, and (**d**) Lm γ1. Different elements constituting the structures are labeled in the figure. Disulfide bridges are displayed in yellow.

BM are cell-adherent extracellular matrices (ECM) essential for cell signaling, polarization, differentiation, and maintenance of tissue organization^1,2^. BM are composed of Lm, type IV collagen, proteoglycans, and nidogens. In mammals, there are five Lm α, four β, three γ, and two splice variants (α3A, α3B), which assemble into functional heterotrimers in at least sixteen different combinations in a tissue-dependent manner. Each Lm isoform is an elongated multidomain glycoprotein comprised of the N-terminal LN domain with the seven- or eight-stranded antiparallel β-sheet jelly roll motif, followed by a rod-like tandem of random-coil LE domains containing the EGF-like fold, an extended α-helical region, and a cluster of globular domains that bind to cell surface receptors at the C-terminus^3^. The heterotrimeric Lm complex is assembled upon joining the α-helical regions from α, β, and γ subunits to form the coiled-coil, also known as the long arm, and producing three N-terminal short arms, each consisting of one LN and multiple LE domains (Fig. 1a). A network of Lm heterotrimers forms a planar lattice on cell surfaces^4^. The Lm polymer node is the basic repeating unit of the lattice containing one of each α, β and γ short arms (Fig. 1b) ^5^. Crystal structures of the N-terminal fragments from individual α5^6^, β1^7^, and γ1^7^ isoforms have been determined. However, due to its intrinsic flexibility, the trimeric Lm complex has been resistant to crystallization. Recent advances in cryo-EM provide unprecedented insights into structures of dynamic macromolecular complexes^8–12^. Thus, we have recently employed cryo-EM for determination of the 3.7 Å structure of the trimeric Lm complex containing the N-terminal 56 kDa α1, 64 kDa β1, and 52 kDa γ1 subunits, the first structure of the functional Lm polymer node (Fig. 2a) ^13^. The asymmetric Lm polymer node resembles a triskelion with centrally located LN domains and three rod-like structures projecting outwards, each consisting of one or two LE domains (Fig. 2b).

Lm polymerization is a two-step process mediated by the short arms of α, β, and γ subunits. The β-γ dimerization step is followed by a calcium-dependent aggregation step, in which α associates with the β-γ dimer (Fig. 1b) ^14^. The LN domain in γ uniquely contains a calcium-binding site^7^. The cryo-EM structure revealed that the α1-γ1 interface involves two loops, one from α1, and another from γ1. The loop from γ1 consists of amino acids critical for coordination of a calcium ion (Supplementary Fig. 1). Consequently, we proposed that in the absence of a calcium ion the loop in γ1 is not structured, hence the α1-γ1 interface cannot be formed, explaining the calcium dependence for the assembly of a trimeric Lm polymer node^13^.

Genetic loss of Lm subunits or missense mutations at their N-termini result in a diverse group of human disorders. The vast majority of mutations are lethal. They result in stop codons, which prematurely terminate protein synthesis^15,16^. A subset of genetic alterations lead to missense mutations. In such cases, the altered Lm proteins are synthesized, and they can be secreted to ECM. However, the propensity of altered Lm variants for polymerization into the functional Lm lattice is impaired. While the pathogenic mutations occur in different Lm isoforms, the vast majority of them is localized to the LN domains. Hence, we have recently defined this group of human disorders as LN-lamininopathies^13^. LN-lamininopathies include failures of early differentiation and organogenesis (α1, β1, α5), and diseases manifesting in kidney and eye (β2), skin (β3), or muscle, peripheral nerve, and brain (α2). For example, Poretti-Boltshauser syndrome (α1)^17^ is a disorder that affects motor movement and cognitively impairs the patients. Pierson syndrome (β2)^18^ leads to kidney failure. The Junctional Epidermolysis Bullosa (β3)^19^ is characterized by fragility and easy blistering of the skin and mucous membranes. Mutations in α2 cause two types of muscular dystrophy: Laminin α2 Congenital Muscular Dystrophy (LAMA2-CMD), also known as Merosin-Deficient Congenital Muscular Dystrophy type 1A (MDC1A)^20^, and Limb-Girdle Muscular Dystrophy (LGMD)^21^. Both disorders manifest in severe muscle weakness leading to the inability for independent walking^20^, however LGMD occurs later in life^21^. In addition, disruption of the Lm polymer impedes cancer angiogenesis, and reduces the propensity of cancer cells for metastatic spread^22^. Understanding the molecular basis underlying LN-lamininopathies requires the understanding of the pathogenic Lm polymer nodes’ structures.

AlphaFold2^23^ (AF2), the artificial intelligence (AI) based program that employs the transformer neural network to process information extracted from multiple sequence alignments run against available sequence data basis^24^, has demonstrated an unprecedented level of accuracy in modeling and predicting protein structures with sub-angstrom precision^25–30^. AF2 has been previously successfully applied for prediction of structural effects of mutations, such as multi-residue insertions, deletions and substitutions^26^, and improvements of the experimentally-derived, cryo-EM^29,30^, X-ray^30^ and NMR^27,28^ structures, including side chain refinements. In contrast, current applications of AF2 to problems related to protein folded are limited, largely due to the lack of training data sets representing misfolded and partly-folded structures^31,32^. Computed AF2 models are currently available through RCSB PDB^33^. The fact that Lm are evolutionarily highly conserved group of proteins that share significant sequence identity and structure homology makes them suitable for AF2 analysis. We employed machine learning algorithms implemented in the program AF2^23^ to model structures of fifty-five monomeric and trimeric Lm complexes including structures of twenty-three altered Lm polymer nodes implicated in human disease^34^. We demonstrate that the in silico models display a remarkable accuracy and precision by validating a subset of the computed atomic models against experimentally derived cryo-EM^13^ and X-ray structures^6,7^. We present a comprehensive and systematic structural analysis of twenty-three reported to date mutations underlying Lm disorders in their physiological context of the altered trimeric Lm polymer nodes. For the first time, we provide the detailed mechanistic insight explaining how Lm mutations lead to failures in Lm polymer node assembly. Although Lm mutations manifest in a wide spectrum of human diseases, we discovered that they can be grouped into four distinct classes reflecting their structural roles. Consequently, we recategorize the reported to date LN-lamininopathies into four classes based on their underlying molecular basis, rather than associated clinical onsets. Our findings have the potential to facilitate rational drug design aiming in the treatment of Lm disorders.

## Results and discussion

### Cryo-EM structure of the Lm α1β1γ1 polymer node

We have determined a 3.7 Å structure of the trimeric Lm α1β1γ1 polymer node recently described by Kulczyk et al.^13^ (Fig. 2a). The trimeric Lm complex is stabilized by a network of disulfide and hydrogen bonds, and electrostatic and hydrophobic interactions. Individual subunits are foot-like shaped molecules with regions resembling the heel and the toe, stabilized by the structurally conserved seven or eight-stranded jelly-roll β-sheet in each LN domain (Fig. 2c-e). In the seven-stranded β-sheet from α1, strands β3, β5 and β7 form the front face of the β-sheet, whereas strands β1, β2, β4 and β6 assemble into the back face (Fig. 2c). In the eight-stranded jelly-roll motifs from β1 and γ1, strands β3, β6 and β8 form the front inner face of the β-sheet, while strands β1, β2, β4, β5, β7 form the back outer face (Fig. 2d-e). Hydrophobic cores of α1, β1, and γ1 are sandwiched between front and back faces of their jelly-roll motifs. In addition, all subunits contain three conserved α helices (α1, α2 and α3) inserted between individual β-strands. The toe regions in α1, β1, and γ1 subunits are more structurally diverse with α1 consisting of a two-stranded β-sheet between strands β1 and β2 (Fig. 2c). In addition, toe regions from α1, β1, and γ1 contain a varying number of short α-helices i.e. three, one, and two, respectively (Fig. 2c-e). The structure of the Lm polymer node is stabilized by a network of intra-subunit disulfide bonds. There are twelve disulfide bonds in α1, ten in β1 and twelve in γ1 (Fig. 2c-e). The cryo-EM map reveals the presence of eight extended N-glycans attached to its surface (Fig. 2a). The inter-subunit interfaces are formed by two sets of interacting regions involving LN and LE1 domains. The first set involves loops connecting β1 and β2 strands in β-sheets from neighboring subunits, whereas the second set includes loops linking strands β7 and β8 from one subunit, and the N-terminal region along with one of the loops from the LE1 domain in the neighboring subunit (Supplementary Fig. 2).

### Lm isoforms are structurally homologous

**Figure 3:**
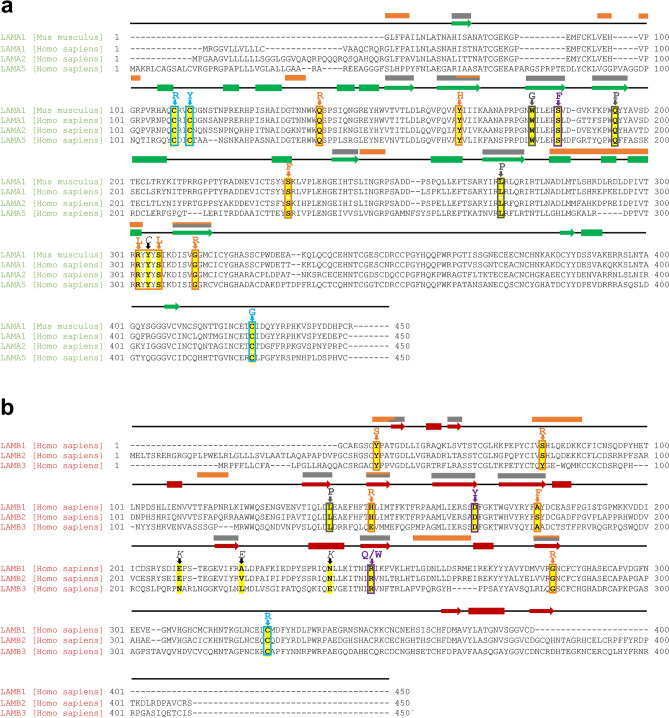
Pathogenic Lm mutations are located in the areas of high sequence conservation. Lm isoforms share significant sequence identity (Supplementary Table 1). We used Clustal Omega^60^ for sequence alignment of the following Lm isoforms implicated in Lm disorders: (**a**) α1 (*Homo sapiens* and *Mus musculus* LAMA1; the mouse Lm α1 was used for cryo-EM structure determination^13^), α2 (LAMA2), α5 (LAMA5), and (**b**) β1 (LAMB1), β2 (LAMB2), and β3 (LAMB3). Class 1, 2, 3 and 4 mutations are conserved among different Lm variants and are indicated by orange, purple, cyan and gray letters, respectively. Secondary structure elements are indicated above amino acid sequences (α-helices as bars and β-strands as arrows in green and red for Lm α and β variants, respectively). Some of the conserved protein regions involved in formation of inter-subunit interfaces and hydrophobic cores are indicated above the amino acid sequences as orange and gray bars, respectively.

**Figure 4:**
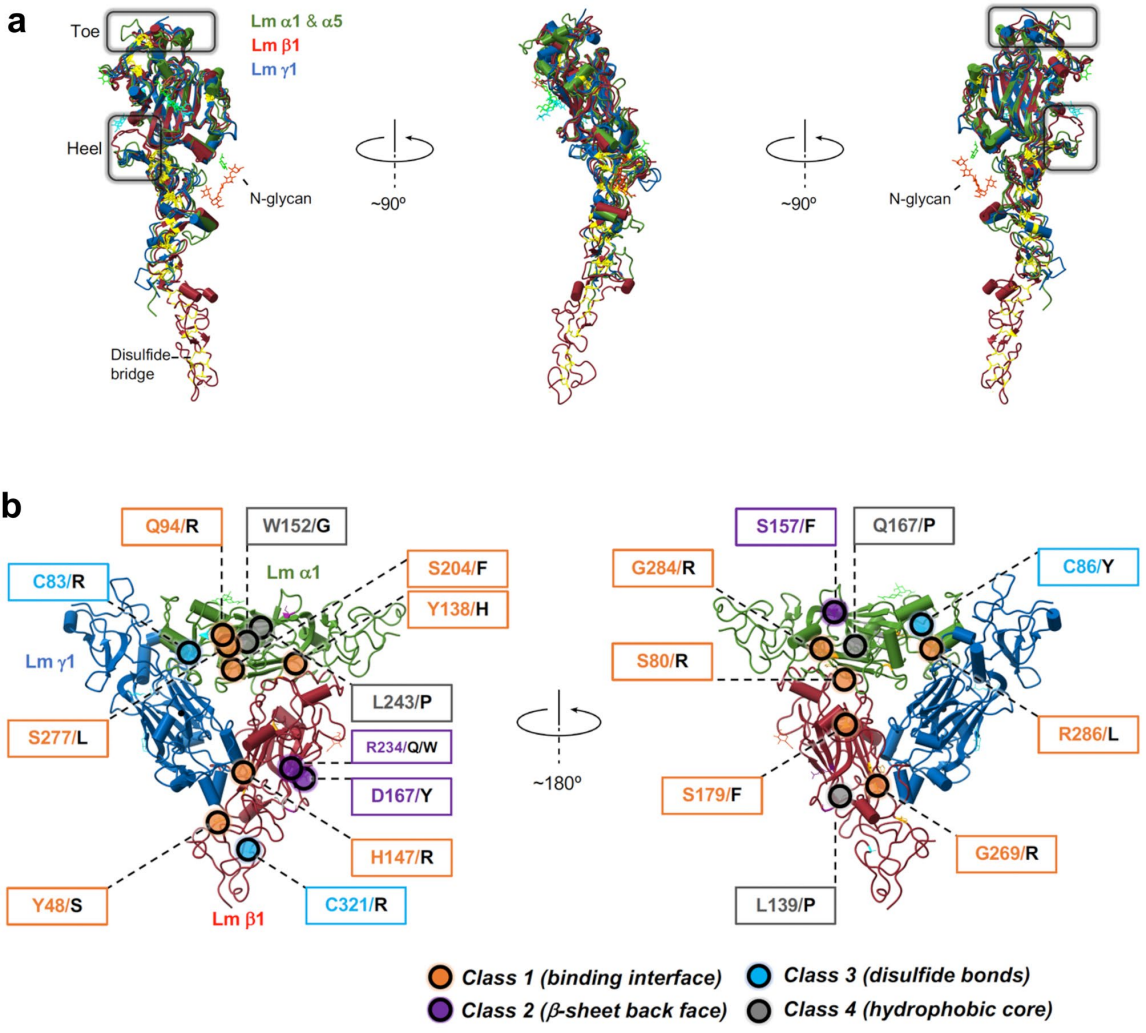
Pathogenic Lm mutations implicated in LN-lamininopathies are located in the areas of high structure homology. (**a**) Lm isoforms share significant structural homology (Supplementary Table 2). Different views of the structures of: α1 (PDB ID: 8DMK^13^), α5 (PDB ID: 2Y38^6^), β1 (PDB IDs: 8DMK^13^ and 4AQS^7^), and γ1 (PDB ID: 4AQT^7^) superposed using the program ChimeraX^63^. Both, Lm α1 and α5 are shown in green. Lm β1 and γ1 are displayed in red and blue, respectively. Lm are evolutionarily conserved group of proteins sharing significant sequence and structure homology, which in conjugation with high accuracy of AF2 predictions (Supplementary Fig. 3, Supplementary Fig. 6, Supplementary Fig. 8) and the agreement of AF2 models with the experimentally-derived structures (Supplementary Fig. 3, Supplementary Fig. 4) provide the basis for the structural analysis of pathogenic Lm polymer nodes with AF2. (**b**) Twenty-three reported to date pathogenic mutations causing LN-lamininopathies^15,34,35^ were mapped onto the cryo-EM structure of the Lm polymer node^13^. Mutations cluster into four distinct structural groups, and they are located in the areas of significant sequence and structure homology. The class 1 mutations (shown in orange) involve residues from inter-subunit binding interfaces. The class 2 mutations (purple) are located in the jelly-roll β-sheet motif in close proximity to invariant N-glycosylation sites. The mutation from the class 3 (shown in cyan) and class 4 (displayed in gray) affect formation of disulfide bridges and hydrophobic cores of altered Lm subunits, respectively.

Lm are a family of evolutionarily highly conserved proteins with individual isoforms sharing an identical domain structure^7,15^. We used Clustal Omega for sequence alignment of the following α and β isoforms: α1, α2, α5 (Fig. 3a), and β1, β2, and β3 (Fig. 3b). These isoforms have been implicated in Lm disorders. The α chains share 52.72–90.49% identity across the LN and LE1 domains (Supplementary Table 1). Likewise, β isoforms share 43.2–71.66% sequence identity (Supplementary Table 1). The vast majority of residues involved in formation of the hydrophobic cores (Fig. 2c) and inter-subunit interfaces (Supplementary Fig. 2) are highly conserved among Lm chains (Fig. 3). In addition, the Lm isoforms share significant structural homology. We have superposed six available atomic models of monomeric Lm α1, α5, β1 and γ1 from the cryo-EM^13^ and X-ray structures^6,7^ (Fig. 4a). The backbone RMSD across all atom pairs from LN and LE1 domains range from 1.59 Å to 5.32 Å (Supplementary Table 2). Significant sequence identity and structure homology, as well as the presence of the experimentally derived structures, make Lm suitable for the analysis with AF2.

### Pathogenic Lm mutations occur in highly conserved regions in Lm structure

Twenty-three pathogenic missense mutations implicated in human disease have been reported to date in polymerizing Lm^15,34,35^. Residues undergoing mutations are highly conserved in the amino acid sequence of Lm isoforms (Fig. 3). Interestingly, all missense mutations occur in Lm α and β isoforms. No mutations have been identified in Lm γ, possibly suggesting that alternations affecting γ subunits are lethal. For the first time, we have mapped these disease causing mutations onto the cryo-EM structure of the Lm polymer node^13^ (Fig. 4b), and discovered that they cluster into four distinct classes located in structurally highly conserved regions (Fig. 4). Consequently, we divided the disease-causing mutations into four classes reflecting structural roles that they play in Lm polymer node assembly (Supplementary Table 3).

In addition to twenty-three amino-acid alternations affecting polymerizing Lm subunis^15,34,35^, another mutation (Glu 210/Lys) implicated in Junctional Epidermolysis Bullosa^19^ was detected in a non-polymerizing^36^ Lm β3. Furthermore, three amino acid substitutions were identified in other species, namely Tyr 258/Cys in *Mus musculus* α1^37^, and Glu198/Lys and Ala209/Glu in *Drosophila melanogaster* β1^38^ (Supplementary Table 3). These four additional mutations are not discussed in the current article.

In summary, we discovered that mutations underlying LN-lamininopathies populate four structurally distinct, yet conserved regions in Lm structure, the latter fact further supporting the applicability of AF2 to study pathogenic Lm mutations.

### Structural modeling of monomeric and trimeric Lm complexes with AF2 and validation of resultant models against experimentally derived structures

To determine applicability of AF2 for prediction of Lm structures, we have employed AF2 equipped with the sequence search module MMseq2^23^ and AMBER energy minimization for structural modeling of four monomeric Lm isoforms and a trimeric Lm α1β1γ1 polymer node, whose experimentally-derived structures are available^6,7,13^. We first generated structural models of monomeric Lm α1^13^, α5^6^, β1^7,13^ and γ1^7,13^, and compared these models with existent cryo-EM^13^ and X-ray structures (Supplementary Fig. 3). The predicted models are in excellent agreement with structures derived experimentally. The backbone RMSDs calculated for the following pairs of AF2 model vs. experimental structure are: 1.12 Å for α5 (333 residues, PDB ID: 2Y38), 1.93 Å for β1 (323 residues, PDB ID: 4AQS), 1.56 Å for γ1 (356 residues, PDB ID: 4AQT), 2.97 Å across 305 residues for α1 (PDB ID: 8DMK), 1.58 Å for β1 (306 residues, PDB ID: 8DMK), 2 Å for γ1 (303 residues, PDB ID: 8DMK). All residues from the experimentally-derived models were used for RMSD calculations. The accuracy of AF2 predictions is reflected by high Predicted Local Distance Difference Test (pLDDT) values with the baseline of approximately 98 for all modeled Lm chains (Supplementary Fig. 3c). Although some of the protein loops from structures of Lm monomers are modeled with less confidence i.e. pLDDT values ranging from 49 to 98 (Supplementary Fig. 3c), the conformation of these loops have been verified against experimentally derived structures (Supplementary Fig. 3a, b), the fact reflected by low RMSD values calculated between the in silico and experimentally derived models. In particular, the cryo-EM structure of Lm α1β1γ1^13^ provides the guide allowing for verification of local structures in these regions in the context of the trimeric Lm polymer node complex (Supplementary Fig. 3a). Importantly, only one out of twenty-three Lm mutations underlying LN-lamininopathies, namely β2(S80R), is located in these regions modeled with lower confidence. The β2(S80R) was previously extensively investigated biochemically^39^ and structurally^13^. The AF2 model of α5β2(S80R)γ1 and the cryo-EM structure of Lm α1β1γ are in good agreement in the region containing the mutation (Supplementary Fig. 15). Furthermore, it was shown experimentally that this mutation disrupts a trimeric structure of the Lm polymer node^39^, confirming our structural analysis described in the section concerned with mutations affecting the inter-subunit binding interfaces within Lm polymer nodes.

**Figure 5:**
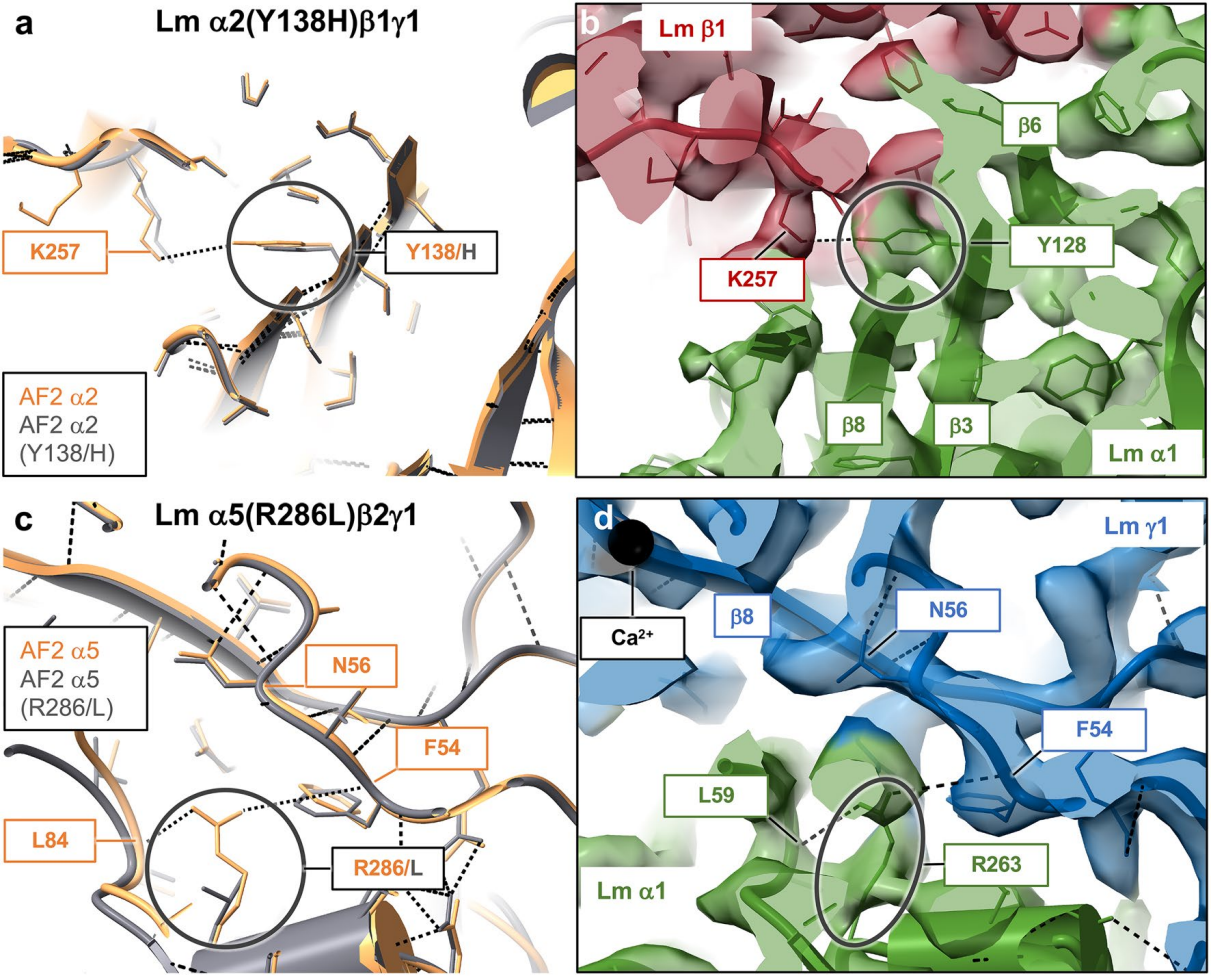
Mutations affecting the inter-subunit binding interfaces in Lm polymer nodes (class 1). (**a**) A substitution of Tyr 138 in α2 with histidine breaks the hydrogen bond formed by tyrosine with Lys 257 from β1, affecting the α2-β1 interface and consequently disrupting a trimeric structure of the Lm α2(Y138H)β1γ polymer node. The Tyr 138/His mutation leads to LAMA2-CMD^47^. (**b**) The cryo-EM structure of Lm α1β1γ1^13^ revels an analogous hydrogen bond formed by Tyr 128 from α1 and Lys 257 from β1. (**c**) A mutation of Arg 286 to leucine in α5(R286L)β2γ1causes developmental disorders of kidney, face, and limbs^44^ by disrupting a network of hydrogen bonds stabilizing the α5-γ1 interface, namely an inter-subunit bond formed by Arg 286 with Phe 54 from γ1, and intra-subunit hydrogen bond formed by Arg 286 with Leu 84. (**d**) Analogous hydrogen bonding pattern is present in the cryo-EM structure of Lm α1β1γ1^13^, and it involves Arg 263 and Leu 59 from α1, and Phe 54 from γ1. AF2 models of wild-type and altered polymer nodes are displayed in orange and gray, respectively. The cryo-EM structure of Lm α1β1γ1^13^ is color-coded with α1, β1 and γ1 shown in green, red and blue, respectively. Previous site-directed mutagenesis of α1(Y128R)^5^, α1(R263D)^5^ and SEC fractionation of Lm oligomers reconstituted with genetically-altered subunits, revealed that these two mutations disrupt formation of the trimeric Lm polymer nodes, consistent with our structural AF2 analysis.

**Figure 6:**
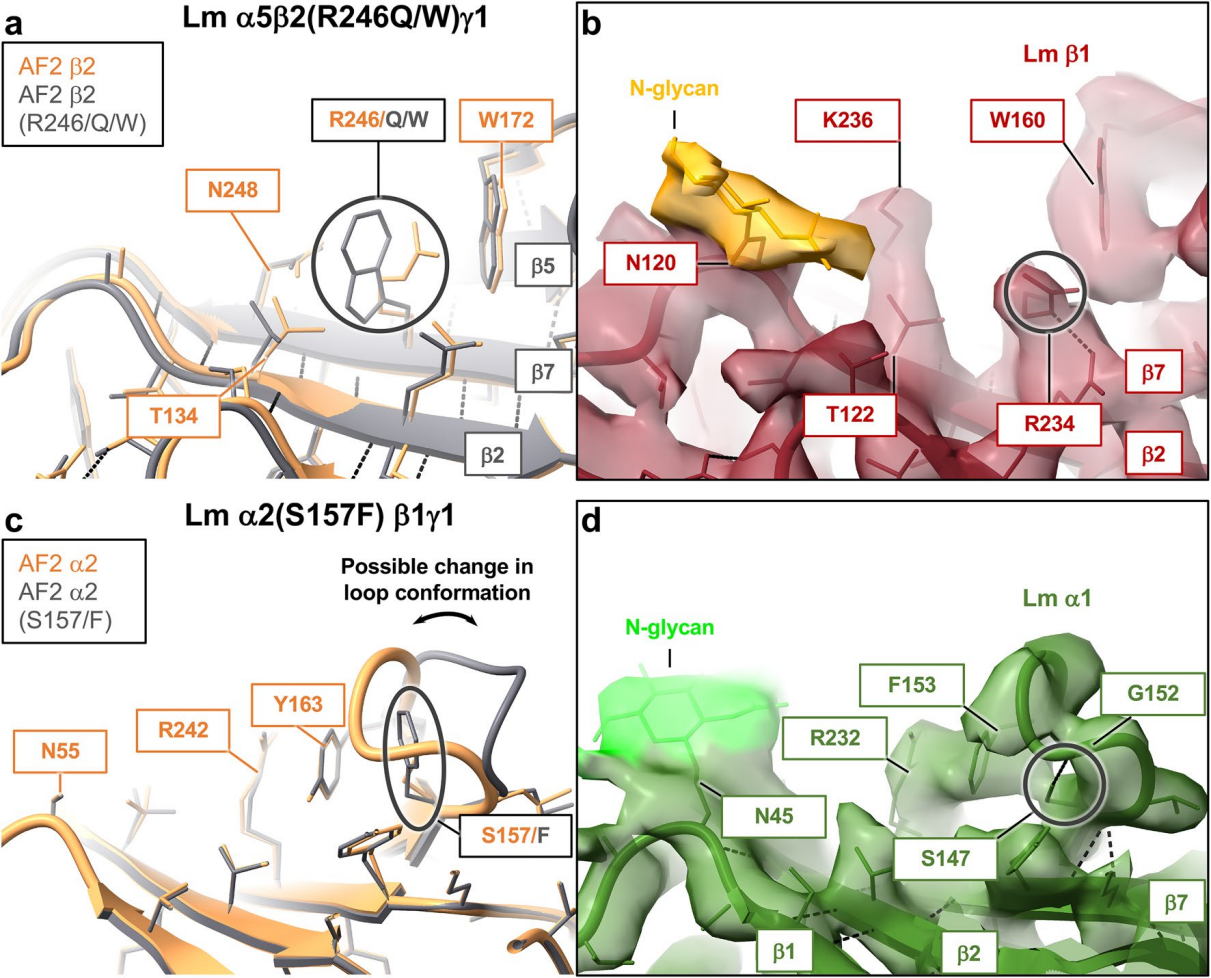
Mutations located in close proximity to the N-glycosylation sites on the back face of the jelly-roll motifs in Lm subunits (class 2). (**a**) Arg 246/Gln^18^ and Arg 246/Trp^50^ are frequent mutations producing severe phenotypes of Pierson syndrome^42^. Arg 246 is positioned on the back face of the β-sheet in the location adjacent to an invariant N-glycosylation site, Asn 248. Substitutions of a positively charged Arg 246 with a neutral glutamine or an indole ring of tryptophan destabilize the short loop connecting strands β4 and β5 through possible stacking interactions with the neighboring Trp 172, and may also interfere with N-glycosylation of β2. (**b**) The cryo-EM structure of Lm α1β1γ1 revealed a similar arrangement of side chains in this region with analogous Arg 234, an invariant N-glycosylation site involving Asn 120 and Trp 160 from the surface loop connecting β4 and β5 strands, all located in close proximity to one another (**c**) Ser 157 is positioned on the outer surface of α2’s jelly-roll β-sheet in location adjacent to the invariant N-glycosylation site, Asn 55. Substitution of a Ser 157 with the aromatic ring of phenylalanine likely affects the conformation of a short surface loop connecting strands β4 and β5 with an overall negative effect on protein stability. The Ser 157/Phe mutation in α2(S157F)β1γ1 causes LAMA2-CMD^48^. (**d**) Likewise, in the cryo-EM structure of Lm α1β1γ1^13^ an analogous Ser 147 forms a hydrogen bond with Gly 152 stabilizing a short loop connecting strands β4 and β5 in the jelly-roll motif of α1.

**Figure 7:**
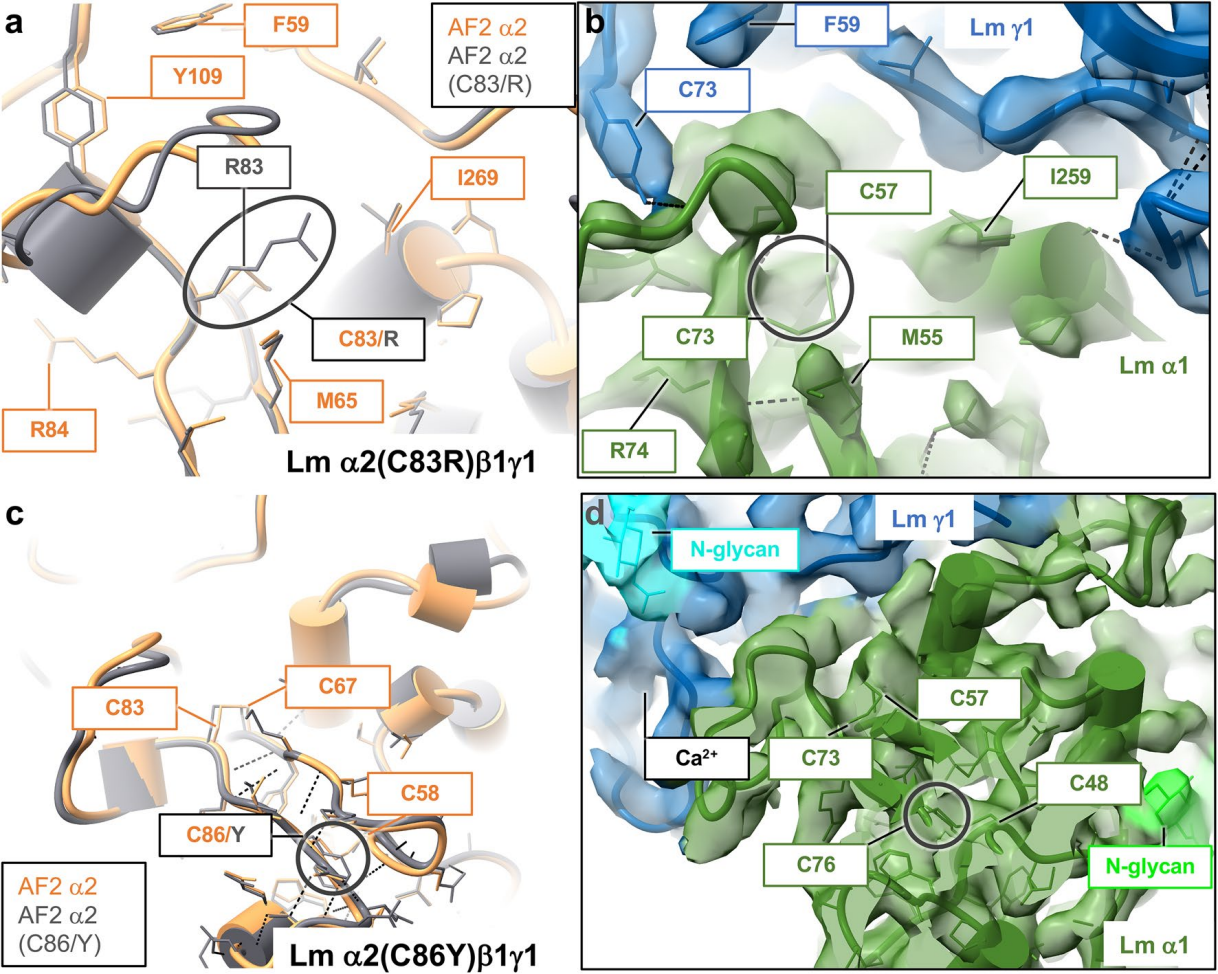
Mutations disrupting disulfide bridges, which stabilize structures of Lm subunits (class 3). (**a**) A substitution of Cys 83 with arginine in α2(C83R)β1γ1 breaks a disulfide bridge formed by Cys 83 and Cys 67, which destabilizes the structure of α2’s toe region involved in formation of the α2-γ1 binding interface. (**b**) In the cryo-EM structure of Lm α1β1γ1^13^, the disulfide bond formed by Cys 73 and Cys 57 plays an identical role in stabilization of the toe region of α1. (**c**) Likewise, the disulfide bond between Cys 58 and Cys C86 constrains the structure of the toe region in α2. The Cys 86/Gly^1^ mutation destabilizes the toe and the α2-γ1 interface. (**d**) A disulfide bridge connecting Cys 48 and Cys 76 plays analogous role in the cryo-EM structure of Lm α1β1γ1^13^. Both Cys83/Arg^55^ and Cys86/Gly^1^ substitutions cause LAMA2-CMD^20,55^. AF2 models of wild-type and altered polymer nodes are displayed in orange and gray, respectively. The cryo-EM structure of Lm α1β1γ1^13^ is color-coded with α1, β1 and γ1 shown in green, red and blue, respectively.

**Figure 8:**
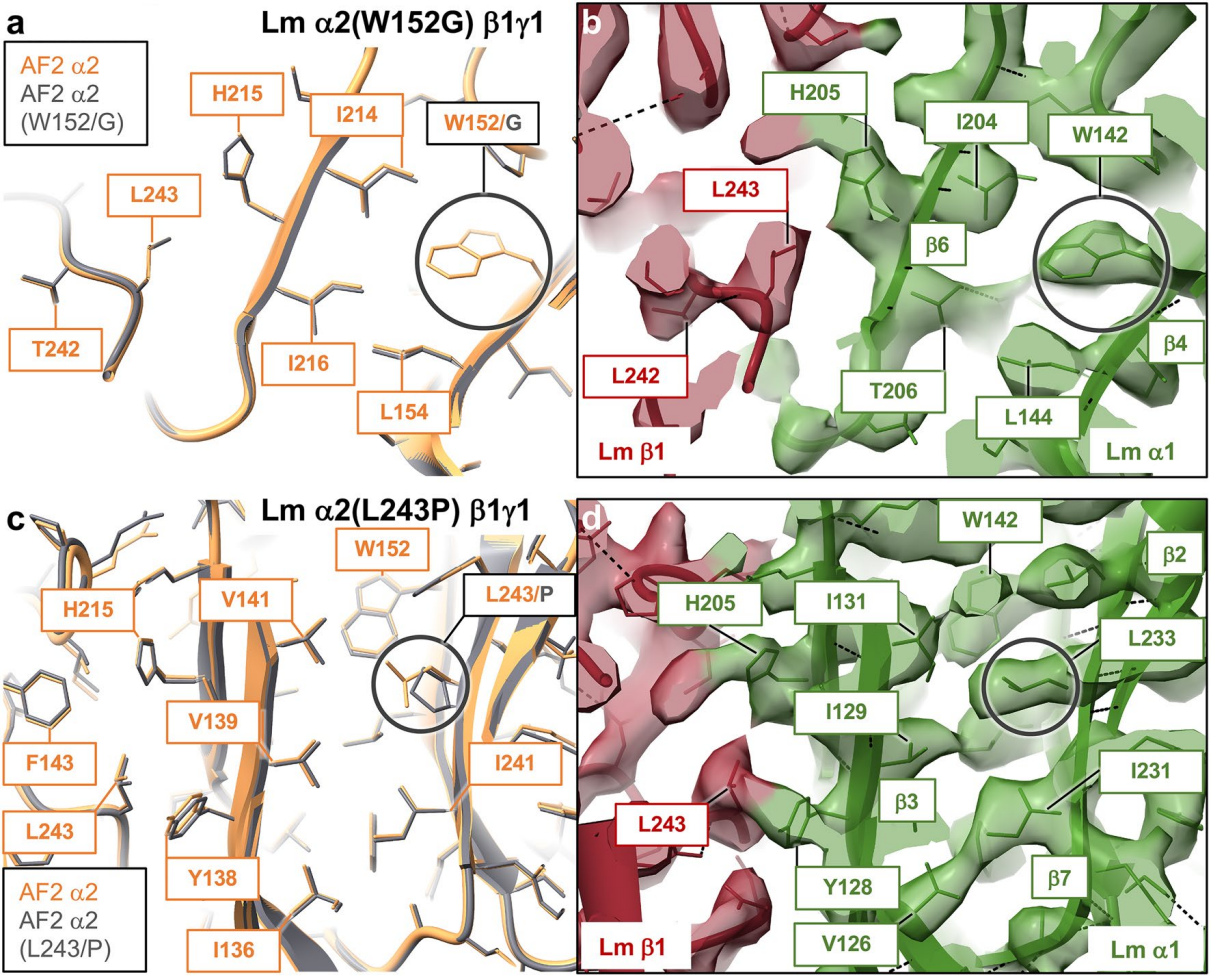
Mutations affecting hydrophobic cores of Lm subunits (class 4). (**a**) A substitution of Trp 152 with glycine in α2(W152G)β1γ1 affects hydrophobic interactions formed by Trp 152 with neighboring residues Leu 154, Ile 214 and Ile 216, which may lead to misfolding of α2. (**b**) The cryo-EM structure of Lm α1β1γ1^13^ reveals a similar network of amino acids forming a hydrophobic core of α1 (Trp 142, Leu 144, and Ile 204). (**c**) Similarly, a mutation of Leu 243 to proline in α2 disturbs a network of interactions involving Val 139, Val 141 and Ile 241, which stabilize the hydrophobic core of the subunit, and indirectly the α2-β1 interface. (**d**) Analogous residues form a hydrophobic core of α1 (Ile 129, Ile 131, Ile 231 and Leu 233) in the cryo-EM structure of Lm α1β1γ1^13^. The Trp 152/Gly and Leu 243/Pro mutations lead to the development of LGMD^21,58^. AF2 models of wild-type and altered polymer nodes are displayed in orange and gray, respectively. The cryo-EM structure of Lm α1β1γ1^13^ is color-coded with α1, β1 and γ1 shown in green, red and blue, respectively.

In the next step, we used AF2 for structural modeling of the trimeric Lm polymer node. The cryo-EM structure of Lm α1β1γ1^13^ is the first structure of any Lm polymer node reported to date, hence we employed the molecular model derived from the cryo-EM Coulomb map as a template to assess the accuracy of multichain AF2 predictions. The map’s nominal resolution of 3.7 Å and local resolutions reaching 3.2 Å allowed for the assignment of side chain rotamers. In addition, the experimentally derived molecular model of Lm α1β1γ1^13^ was not included in the AF2 training data set. A superposition of the experimental structure (PDB ID: 8DMK) with the AF2 model of Lm α1β1γ1 is presented in Supplementary Fig. 4. Our analysis reveals an excellent agreement between the predicted and experimentally derived molecular models with the backbone RMSDs of 0.94 Å and 2.4 Å across LN (274 residues), and LN and LE1 (domains 305 residues), respectively. The RMSDs calculated for all atom pairs including side chain atoms but excluding hydrogens from the LN domains are 2.7 Å for α1 (2002 atom pairs), 2.5 Å for β1 (2454 atom pairs), and 1.96 Å for γ1 (2391 atom pairs). Analogous RMSDs calculated for LN and LE1 domains are 4.2 Å for α1 (4764 atom pairs), 3.68 Å for β1 (4795 atom pairs), and 3.61 Å for γ1 (4604 atom pairs). Because some of the side chains in protein structure are better refined than others, it is important to note that the calculated RMSDs statistically reflect all side chains. Importantly, our results confirm the agreement in side chain rotamers between experimentally and computationally derived molecular models in protein regions, which are a subject of the in silico analysis presented below (Fig. 5, 6, 7, 8 and Supplementary Figs. 9–21). The results prove the accuracy and precision of the Lm α1β1γ1 model generated with AF2. This fact is important for our subsequent analysis of altered Lm polymer nodes containing single point mutations. Accuracy of AF2 predictions is reflected by high pLDDT values with the baseline at approximately 98 (Supplementary Fig. 8). As mentioned above, although some of the loops are modeled with pLDDT values ranging from 38 to 98 (Supplementary Fig. 8), conformations of these loops have been modelled correctly, as they are in similar positions as loops from the experimentally-derived structure of Lm α1β1γ1 (Supplementary Fig. 4), the notion also supported by low RMSD values calculated between the in silico and the experimentally derived models. Although, AF2 is currently unable to predict binding of small ligands, such as a calcium ion present in the cryo-EM structure of Lm α1β1γ1 polymer node^13^, the protein regions implicated in calcium binding (namely the γ1 loop containing residues L106-T116 and the α1-γ1 interface involving the aforementioned γ1 loop and another loop from α1 spanning residues K58-Q72, Supplementary Fig. 1) adopt analogous conformations in both, the AF2 model and the cryo-EM structure, with an overall RMSD of 2.4 Å calculated for all atoms (Supplementary Fig. 4). In addition, there are no pathogenic mutations located in these regions.

Taken together, we have demonstrated in a quantitative fashion that the computed AF2 models of Lm monomers and a trimer are in excellent agreement with models derived experimentally. Thus, we have established AF2 as a viable tool for structure modeling and prediction of pathogenic Lm polymer nodes.

### Structural modeling of pathogenic Lm monomers and polymer nodes with AF2

Having demonstrated that AF2 can be used for accurate structure prediction of monomeric and trimeric Lm complexes, we modeled structures of wild-type α2 and β2, and twenty-three monomeric Lm isoforms harboring pathogenic mutations. Structural models, along with corresponding pLDDT plots, are presented in Supplementary Fig. 5 and Supplementary Fig. 6, respectively. Accuracy of AF2 predictions is reflected by high pLDDT values with the baseline at approximately 98, comparable to those pLDDT values obtained for AF2 models validated against experimentally derived structures (Supplementary Fig. 3c).

Next, we modeled structures of trimeric wild-type Lm α2β1γ1 and Lm α5β2γ polymer nodes, along with twenty-three Lm polymer node structures containing pathogenic mutations. The resultant models and the corresponding pLDDT plots are presented in Supplementary Fig. 7 and Supplementary Fig. 8, respectively. The pLDDT plots, with the baseline at approximately 98, confirm the accuracy of AF2 predictions. AF2 models of pathogenic Lm polymer nodes reveal an extraordinary structure conservation, with RMSDs calculated for all atom pairs from the LN domains harboring mutations ranging from 1.18 Å to 1.58 Å, and in the range of 0.81–0.86 Å for pruned atom pairs (Supplementary Table 4). The LE1 rods are slightly more diverged with RMSDs ranging from 1.21 Å to 2.16 Å for all atom pairs, and from 0.83 Å to 1.06 Å for pruned atom pairs, calculated for both the LN and LE1 domains (Supplementary Table 4). A careful analysis of AF2 models of altered and wild-type Lm polymer nodes in the regions consisting of pathogenic mutations, and the inspection of the corresponded positions in the experimentally-derived cryo-EM structure of Lm α1β1γ1^13^ (Figs. 5, 6, 7, 8 and Supplementary Figs. 9–21) revealed a remarkable structural homology with conserved sidechains adopting identical or nearly identical positions, providing the basis for our structural analysis.

In summary, we modeled fifty-five monomeric and trimeric Lm complexes including twenty-three altered Lm polymer nodes implicated in LN-lamininopathies (Supplementary Table 3). In the following sections we present the detailed structural analysis, which reveals the molecular basis underlying LN-lamininopathies. Due to the current limitation of AF2 for predicting point mutations resulting in defective protein folding^31,32^, our analysis is focused on local effects that pathogenic mutations incur on Lm polymer node structure, such as changes in local hydrogen bond patterns. We recategorize all identified to date Lm disorders into four distinct functional classes based on their underlying mechanistic defects, rather than phenotypic onsets.

### Mutations affecting the inter-subunit binding interfaces in Lm polymer nodes (class 1)

Eleven class 1 mutations are located at the inter-subunit interfaces (Fig. 4b) formed by altered α1, α2, α5, β1, and β2 (Supplementary Table 3) in the pathogenic Lm polymer nodes α1(Q94R)β1γ1, α2(Y138H)β1γ1, α2(S204F)β1γ1, α2(S277L)β1γ1, α2(G284R)β1γ1, α2β1(G269R)γ1, (α5β2(Y48S)γ1, α5β2(S80R)γ1, α5β2(H147R)γ1, α5β2(S179F)γ1, and α5(R286L)β2γ1 (Supplementary Table 4). The class 1 mutations affect Lm propensity for oligomerization by interfering with formation of the α-β (three mutations), α-γ and β-γ interfaces (4 mutations each)^39^. In the majority of cases the aforementioned amino-acid alternations disrupt the network of hydrogen bonds stabilizing the neighboring subunits within the trimer while displaying the destabilizing effect on Lm polymer nodes, as evidenced by changes in calculated ΔΔG values (Supplementary Table 5). Although altered Lm polymer nodes from this class share the common molecular defect, they manifest in a wide spectrum of different human disorders, including LAMA2-CMD^17^, LGMD^40^, Pierson syndrome^40–43^, Poretti-Boltshauser syndrome^17^, and developmental disorders of kidney, face, limbs^44^, and heart^38^ (Supplementary Table 3). The differences in disease manifestation are due to the tissue-specific expression patterns of Lm chains at various developmental stages^45^. For instance, Lm α1 is ubiquitously expressed in the embryonic stage, but its level of expression progressively decreases during development. Consequently Lm α1β1γ1 is restricted to a small subset of BMs. In contrast, Lm α5β2γ1 and Lm α2β1γ1 are the most ubiquitous isoforms present in the adults^46^. Lm α2β1γ1 is mainly expressed in skeletal and cardiac muscles, while Lm α5β2γ1 is expressed in heart kidney and lungs^46^.

The amino-acid substitution of Tyr 138 with histidine in α2(Y138H)β1γ1, implicated in LAMA2-CMD^47^, disrupts the hydrogen bond formed by Tyr 138 and Lys 257 from β1, consequently affecting formation of the α2-β1 interface (Fig. 5b). In the cryo-EM structure of Lm α1β1γ1^13^ an analogous hydrogen bond is formed by Tyr 128 from α1 and Lys 257 from β1 (Fig. 5a). Another example of the mutation from this group is a substitution of Arg 286 to leucine in α5(R286L)β2γ1 causing developmental disorders of kidney, face, and limbs^44^ in children by disrupting a network of neighboring hydrogen bonds stabilizing the α5-γ1 interface. The AF2 model of α5(R286L)β2γ1 reveals that Arg 286 interacts with Phe 54 from β2, and with Leu 84 from α5 (Fig. 5c). Analogous hydrogen bonding interactions of Arg 263 with Phe 54 and Leu 59 are present in the cryo-EM structure of Lm α1β1γ1^13^ (Fig. 5d).

Previous site-directed mutagenesis of α1(Y128R)^5^, α1(R263D)^5^ and other residues from the class 1^39^, followed by Size Exclusion Chromatography (SEC) fractionation of Lm oligomers reconstituted with genetically-altered subunits, revealed that these mutations disrupt formation of the trimeric Lm polymer nodes, consistent with our structural AF2 analysis. Structural effects of the remaining nine class 1 mutations on the following pathogenic Lm polymer nodes: α1(Q94R)β1γ1, α2(S204F)β1γ1, α2(S277L)β1γ1, α2(G284R)β1γ1, α2β1(G269R)γ1, (α5β2(Y48S)γ1, α5β2(S80R)γ1, α5β2(H147R)γ1, and α5β2(S179F)γ1, are discussed in the Supplementary Discussion and presented in the Supplementary Figs. 9–17.

### Mutations located in close proximity to the N-glycosylation sites on the back face of the jelly-roll motifs in Lm subunits (class 2)

In the class 2 there are four amino acid substitutions located at the β-sheet’s back face of α2 and β2 subunits (Supplementary Table 3) in pathogenic Lm polymer nodes α2(S157F)β1γ1, α5β2(D167Y)γ1, α5β2(R246Q)γ1 and α5β2(R246W)γ1 (Supplementary Table 4), causing LAMA2-CMD^48^ and Pierson syndrome^18,49,50^. The cryo-EM map of Lm α1β1γ1^13^ unveiled the presence of extended densities attached to its surface (Fig. 2a). The MS glycopeptide and the released glycan analyses confirmed that these extended densities represent eight unique N-glycans colocalizing with protrusions in the 3D Coulomb map^13^. It has been previously suggested that the presence of N-glycans on the surface of Lm subunits is important for protein oligomerization and Lm polymer node formation in vivo ^51^. Mutations from this class disrupt the local Lm conformation with an overall negative effect on protein stability (Supplementary Table 5), and may alter the N-glycosylation pattern of Lm subunits. Interestingly, recent studies indicated that changes in the N-glycosylation pattern of Lm lattice correlate with the propensity of cancer cells for metastasis^51^.

The Arg 246/Gln^18^ and Arg 246/Trp^50^ are frequent mutations producing severe phenotypes of Pierson syndrome^42^. Arg 246 is positioned at the back face of the β-sheet in the location adjacent to Asn 248 (~ 3 Å away) from the conserved Asn 248–Leu 249–Thr 250 motif (Fig. 6a). Due to close proximity of aforementioned mutations to an invariant N-glycosylation site^52,53^, substitutions of a positively charged Arg 246 with a neutral glutamine or an indole ring of tryptophan, may interfere with N-glycosylation of β2 by oligosaccharyltransferase. In addition, Trp 246 may form stacking interactions with Trp 172 while destabilizing the short loop connecting strands β4 and β5, subsequently leading to an overall unfavorable effect on Lm polymer node’s stability (Supplementary Table 5). The cryo-EM structure of Lm α1β1γ1 reveals a nearly identical arrangement of side chains in this region, with analogous Arg 234 and invariant Asn 120. However, Asn 120 is located on the neighboring β-strands (Fig. 6b).

A substitution of Ser 157 to phenylalanine in α2(S157F)β1γ1 results in LAMA2-CMD^48^ (Fig. 6c). In the cryo-EM structure of Lm α1β1γ1^13^, analogous Ser 147 forms a hydrogen bond with Gly 152 (Fig. 6d). This interaction stabilizes a short loop spanning residues Ser 147-Phe 153 inserted between strands β4 and β5 at the back face of the jelly-roll β-sheet motif. Likewise, in α2(S157F)β1γ1, Ser 157 stabilizes the loop spanning residues Phe 157-Tyr 163. The mutation of serine to phenylalanine likely changes the conformation of this loop, which in turn negatively impacts protein stability evidenced by the change in calculated ΔΔG (Supplementary Table 5). Side-directed mutagenesis of residues from the surface of β-sheets, and they substitutions with aromatics have been shown to destabilize protein structures^54^. Structural effects of β2(D167Y) mutation in α5β2(D167Y)γ1 polymer node are discussed in the Supplementary Discussion and presented in the Supplementary Fig. 18.

### Mutations disrupting formation of disulfide bonds in Lm subunits (class 3)

There are four amino acid mutations in this class. They interfere in formation of specific disulfide bridges in α2 and β2 (Supplementary Table 3), which stabilize structures of α2(C83R)β1γ1, α2(C86Y)β1γ1, α5β2(C321R)γ1, and α2(C393G)β1γ1 polymer nodes (Supplementary Table 4). Lm subunits contain a significant content of random coil loops, which structures are constrained by multiple disulfide bridges. Ten disulfide bonds in β1 and twelve disulfide bonds in each α1 and γ1 stabilize the structure of Lm α1β1γ1 polymer node visualized by cryo-EM^13^. A network of disulfide bridges is displayed in yellow in Fig. 2a, c-e. The class 3 mutations have been implicated in a diverse spectrum of human disorders including LAMA2-CMD^20,55^, LGMD^56^ and Pierson syndrome^57^.

Both, Cys 83/Arg^55^ and Cys86/Gly^1^ substitutions in α2 of Lm α2β1γ1 cause LAMA2-CMD^20,55^ by disrupting a structure of the toe region (Fig. 2c) implicated in formation of the α2-γ1 binding interface (Fig. 7). Cys 83 and Cys 67 form a disulfide bridge connecting the two-stranded β-sheet, which constrains the conformation of the flexible loop 1 in α2 (Supplementary Fig. 3) for its interaction with γ1 (Supplementary Fig. 1). The replacement of Cys 83 with arginine most likely destabilizes the structure in this region while disrupting the α2-γ1 interface (Fig. 7a). In the cryo-EM structure, a disulfide bond formed by Cys 73 and Cys 57 plays the same role in stabilization of the toe region of α1 (Fig. 7b). Likewise, a disulfide bridge linking Cys 58 and Cys 86 preserves the structure of the toe region in α2. The Cys 86/Gly^1^ mutation most likely destabilizes the structure of the toe (Supplementary Table 5), subsequently affecting the α2-γ1 interface (Fig. 7c). A similar interaction involving α1’s Cys 48 and Cys 76 was revealed by the cryo-EM structure of Lm α1β1γ1^13^ (Fig. 7d). The effects of α2(C393G) and β2(C321R) mutations on structures of Lm α2(C393G)β1γ1 and α5β2(C321R)γ1 polymer nodes are discussed in the Supplementary Discussion and presented in the Supplementary Fig. 19.

### Mutations affecting hydrophobic cores of Lm subunits (class 4)

Four class 3 mutations disrupt hydrophobic cores of α2 and β2 (Supplementary Table 3) in pathogenic Lm polymer nodes α2(W152G)β1γ1, α2(Q167P)β1γ1, α2(L243P)β1γ1 and α5β2(L139P)γ1 (Supplementary Table 4), leading to LGMD^21,58^ and Pierson syndrome^42^. Interestingly, hydrophobic cores in α and β subunits exhibit a remarkable structural conservation with RMSDs calculated for pruned atom pairs from the LN domains containing these residues ranging from 0.81 Å to 0.86 Å, indicative of the importance of Lm hydrophobic cores in the maintenance of the Lm polymer nodes’ architecture (Supplementary Table 4).

The Trp 152/Gly and Leu 243/Pro mutations in α2β1γ1 cause LGMD^21,58^ by destabilizing the hydrophobic core of the jelly-roll motif in α2 (Supplementary Table 5), which in turn may affect formation of the α2-β1 binding interface. A substitution of Trp 152 with glycine affects hydrophobic interactions formed by Trp 152 with neighboring Leu 154, Ile 214 and Ile 216 (Fig. 8a). The cryo-EM structure reveals a similar network of interplaying residues, including Trp 142, Leu 144, and Ile 204 (Fig. 8b). Likewise, a mutation of Leu 243 to proline disturbs a network of interactions involving Val 139, Val 141 and Ile 241 (Fig. 8c). The cryo-EM structure of Lm α1β1γ1^13^ revealed that analogous amino acids (Ile 129, Ile 131, Ile 231 and Leu 233) form a hydrophobic core of α1, (Fig. 8d). It is conceivable that point mutations within the hydrophobic cores of Lm subunits may lead to defective protein folding^31,32^, however such global structural changes would likely not be detected by the current version of AF2. For instance, prolines have a low propensity for β-sheet formation^59^, hence Leu 243/Pro mutation in α2β1γ1 may result in larger structural changes than AF2 predicted. Structural effects of α2(Q167P) and β2(L139P) mutations in Lm polymer nodes α2(Q167P)β1γ1and α5β2(L139P)γ1 are discussed in the Supplementary Discussion, and they are presented in Supplementary Figs. 20–21.

### Molecular basis underlying LN-lamininopathies

We have employed cryo-EM and AF2 to analyze molecular basis of LN-lamininopathies. Although, LN-lamininopathies are a diverse group of human disorders resulting in a wide spectrum of medical onsets, these disorders have common mechanistic basis. We have carefully analyzed local perturbations in Lm structure incurred by a set of twenty-three identified to date single amino acid substitutions causing LN-lamininopathies. In contrast, such an analysis cannot be conducted with the aid of previously determined X-ray structures of monomeric Lm chains^6,7^, because the vast majority of mutations affect formation of inter-subunit interfaces that are absent from the crystal structures. In addition, some of the flexible loops involved in formation of the inter-subunit interfaces are either missing in the crystal structures or adopt different conformations. Furthermore, unlike the X-ray structures of Lm monomers^6,7^ and the cryo-EM structure of the Lm α1β1γ1 polymer node^13^, the AF2 models allow for structural analysis of Lm mutations in their physiological context of the pathogenic Lm polymer node complexes.

Taken together, we discovered that the Lm mutations can be grouped into four distinct structural classes, namely (i) mutations affecting formation of the inter-subunit binding interfaces in Lm polymer nodes (Fig. 5 and Supplementary Figs. 9–17), (ii) amino acid alternations located in close proximity to the N-glycosylation sites on the back face of the jelly-roll motifs that destabilize the local protein conformation and may interfere with N-glycosylation of Lm subunits (Fig. 6 and Supplementary Fig. 18), (iii) mutations disrupting disulfide bridges, which stabilize Lm subunits (Fig. 7 and Supplementary Fig. 19), and (iv) amino acid substitutions affecting formation of hydrophobic cores in Lm subunits (Fig. 8 and Supplementary Figs. 20–21). For the first time, our structural analysis reveals the molecular mechanisms underlying LN-lamininopathies, and may foster the development of novel therapeutics for the treatment of Lm deficiencies via the design of BMs for tissue implants or structure-based drug design.

## Methods

### Structure predictions and sequence alignments

We employed ColabFold: AlphaFold2 equipped with the sequence search module MMseq2^23^ for structure prediction and modeling of fifty-five monomeric and trimeric Lm complexes using amino acid sequences corresponding to the LN, LE1 and LE2 domains in each protein construct (Supplementary Information). The “alphafold2_ptm” and “alphafold2_multimer_v3” were used for monomer and trimer predictions, respectively. Structural modeling was run using “Mmseqs2_unirev_env” and “unpaired_paired” pairing strategy with a combination of pair sequences from the same species and unpaired MSA. The paired MSA was executed by searching the UniRef100 database and by pairing the best hits sharing the same NCBI taxonomic identifiers. The unpaired MSA was executed by searching the UniRef100 database and the environmental sequences. Five models were generated for each construct with three recycles for monomers and up to twenty recycles for trimers. The final structural models were relaxed using AMBER and ranked by pLDDT for monomers and by (80*ipTM + 20*pTM) for trimers.

The per residue pLDDT coefficients calculated for each monomeric and trimeric model are presented in Supplementary Fig. 6 and Supplementary Fig. 8, respectively. We calculated twenty-nine models of monomeric Lm isoforms, including: six wild-type subunits (α1, α2, α5, β1, β2, and γ1), and twenty-three subunits harboring pathogenic mutations (α1(Q94R), α2(C83R), α2(C86Y), α2(Y138H), α2(W152G), α2(S157F), α2(Q167P), α2(S204F), α2(L243P), α2(G284R), α2(S277L), α2(C393G), α5(R286L), β1(G269R), β2(Y48S), β2(S80R), β2(L139P), β2(H147R), β2(D167Y), β2(S179/F), β2(R246Q), β2(R246W), β2(C321R)). We also modeled twenty-six trimeric Lm polymer nodes, including: three wild-type trimeric complexes (α1β1γ1, α2β1γ1, and α5β2γ1), and twenty-three polymer nodes containing pathogenic mutations (α1(Q94R)β1γ1, α2(C83R)β1γ1, α2(C86Y)β1γ1, α2(Y138H)β1γ1, α2(W152G)β1γ1, α2(S157F)β1γ1, α2(Q167P)β1γ1, α2(S204F)β1γ1, α2(L243P)β1γ1, α2(S277L)β1γ1, α2(G284R)β1γ1, α2(C393G)β1γ1, α2β1(G269R)γ1, α5β2(Y48S)γ1, α5β2(S80R)γ1, α5β2(L139P)γ1, α5β2(H147R)γ1, α5 β2(D167Y)γ1, α5β2(S179F)γ1, α5β2(R246Q)γ1, α5β2(R246W)γ1, α5(R286L)β2γ1, α5β2(C321R)γ1). Sequence alignments were carried out using Clustal Omega 1.2.4^60^ and InterProScan 5.59–91.0^61^. Energetic effects of single point mutations on the stability of pathogenic Lm polymer nodes were calculated with DynaMut2^62^. Structural alignments and analysis were carried out using UCSF ChimeraX^63^ and UCSF Chimera^64^. The following atomic coordinates and cryo-EM Coulomb maps were used for structural analysis: α1β1γ1 (PDB ID: 8DMK^13^ [https://www.wwpdb.org/pdb?id=pdb_00008dmk], and EMDB ID: EMD-27542^13^ [https://www.ebi.ac.uk/emdb/EMD-27542]), α5 (PDB ID: 2Y38^6^ [https://www.wwpdb.org/pdb?id=pdb_00002y38]), β1 (PDB ID: 4AQS^7^ [https://www.wwpdb.org/pdb?id=pdb_00004aqt]), and γ1 (PDB ID: 4AQT^7^ [https://www.wwpdb.org/pdb?id=pdb_00004aqt]).

### Supplementary Information

Supplementary Information.

## Data Availability

The fifty-five structural models of Lm complexes generated in this study were deposited to ModelArchive^65^. The models are available at [https://modelarchive.org/doi/10.5452/ma-kul-lams], and through the RCSB PDB [https://www.rcsb.org] with the following accession codes: the entire data set (ID: ma-kul-lams), α1 (ID: ma-v5ttj), α2 (ID: ma-druea), α5 (ID: ma-ohjco), β1 (ID: ma-efvh2), β2 (ID: ma-t6221), γ1 (ID: ma-ra61r), α1(Q94R) (ID: ma-o5jpt), α2(C83R) (ID: ma-kouys), α2(C86Y) (ID: ma-ygiyq), α2(Y138H) (ID: ma-8r999), α2(W152G) (ID: ma-2wz4g), α2(S157F) (ID: ma-qfce1), α2(Q167P) (ID: ma-tfa51), α2(S204F) (ID: ma-8uyw8), α2(L243P) (ID: ma-6ukz4), α2(G284R) (ID: ma-nefsb), α2(S277L) (ID: ma-6v6zk), α2(C393G) (ID: ma-x85nl), α5(R286L) (ID: ma-ayla5), β1(G269R) (ID: ma-3mw01), β2(Y48S) (ID: ma-pzf7x), β2(S80R) (ID: ma-93ioo), β2(L139P) (ID: ma-qmcs3), β2(H147R) (ID: ma-6270t), β2(D167Y) (ID: ma-pqt8i), β2(S179/F) (ID: ma-yuf2a), β2(R246Q) (ID: ma-06m7t), β2(R246W) (ID: ma-0lg9n), β2(C321R) (ID: ma-iotwv), α1β1γ1 (ID: ma-2xp55), α2β1γ1 (ID: ma-efpjy), α5β2γ1(ID: ma-t5mf7), α1(Q94R)β1γ1 (ID: ma-gxgul), α2(C83R)β1γ1 (ID: ma-dcwst), α2(C86Y)β1γ1 (ID: ma-gwaoc), α2(Y138H)β1γ1 (ID: ma-4yg9k), α2(W152G)β1γ1 (ID: ma-7obo4), α2(S157F)β1γ1 (ID: ma-oov0i), α2(Q167P)β1γ1 (ID: ma-in648), α2(S204F)β1γ1 (ID: ma-7cryl), α2(L243P)β1γ1 (ID: ma-mqk6c), α2(S277L)β1γ1 (ID: ma-b578o), α2(G284R)β1γ1 (ID: ma-3tov9), α2(C393G)β1γ1 (ID: ma-6ycuj), α2β1(G269R)γ1 (ID: ma-nnvre), α5β2(Y48S)γ1 (ID: ma-f5g6h), α5β2(S80R)γ1 (ID: ma-e9zx7), α5β2(L139P)γ1 (ID: ma-z78hd), α5β2(H147R)γ1 (ID: ma-rd7nz), α5 β2(D167Y)γ1 (ID: ma-93i2u), α5β2(S179F)γ1 (ID: ma-3ttc9), α5β2(R246Q)γ1 (ID: ma-00eo9), α5β2(R246W)γ1 (ID: ma-e3lx8), α5(R286L)β2γ1 (ID: ma-udxx4), α5β2(C321R)γ1 (ID: ma-r2s20).
