# Supplementary material for: Artificial intelligence and the analysis of cryo-EM data provide structural insight into the molecular mechanisms underlying LN-lamininopathies
Source: Sci Rep. 2023 Oct 19;13:17825. doi: 10.1038/s41598-023-45200-5 (PMC10587063; doi:10.1038/s41598-023-45200-5)
Supplement: Supplementary file 1 — Supplementary Information. [file 41598_2023_45200_MOESM1_ESM.pdf]

Artificial intelligence and the analysis of cryo-EM data provide structural insight into the molecular mechanisms underlying LN-lamininopathies.

Arkadiusz W. Kulczyk[1,2]

[1]Institute for Quantitative Biomedicine, Rutgers University, 174 Frelinghuysen Road, Piscataway, NJ 08854, USA, [2]Department of Biochemistry & Microbiology, Rutgers University, 75 Lipman Drive, New Brunswick, NJ 08901, USA

Correspondence should be addressed to Arkadiusz Kulczyk (arek.kulczyk@rutgers.edu)

<u>Inventory of Supplementary Information:</u>

**Supplementary Table 1**	Lm isoforms share significant sequence identity.
**Supplementary Table 2**	Lm isoforms are structurally homologous.
**Supplementary Table 3**	Pathogenic Lm mutations.
**Supplementary Table 4**	Lm polymer nodes are structurally conserved.
**Supplementary Table 5**	Energetic effects of single point mutations on the stability of pathogenic Lm polymer nodes.
**Supplementary Figure 1**	Structural details of the α1-γ1 interface in Lm α1β1γ1 polymer node.
**Supplementary Figure 2**	Inter-subunit interactions stabilizing Lm α1β1γ1 polymer node.
**Supplementary Figure 3**	Validation and superposition of monomeric AF2 models with the experimentally derived structures of Lm α1, α5, β1 and γ1.
**Supplementary Figure 4**	Validation and superposition of the trimeric AF2 model with the cryo-EM structure of Lm α1β1γ1.
**Supplementary Figure 5**	AF2 models of wild-type Lm subunits and pathogenic Lm isoforms implicated in LN-lamininopathies.
**Supplementary Figure 6**	The pLDDT plots calculated for the AF2 models of wild-type and pathogenic Lm isoforms implicated in LN-lamininopathies.
**Supplementary Figure 7**	AF2 models of trimeric wild-type Lm polymer nodes and their pathogenic counterparts causing LN-lamininopathies.
**Supplementary Figure 8**	The pLDDT plots calculated for the AF2 models of wild-type and pathogenic Lm polymer nodes.
**Supplementary Figure 9**	Lm α1(Q94R)β1γ1: class 1 mutation causing Poretti-Boltshauser syndrome.
**Supplementary Figure 10**	Lm α2(S204F)β1γ1: class 1 mutation causing limb-girdle muscular dystrophy.
**Supplementary Figure 11**	Lm α2(S277L)β1γ1: class 1 mutation causing laminin α2 congenital muscular dystrophy.
**Supplementary Figure 12**	Lm α2(G284R)β1γ1: class 1 mutation leading to limb-girdle muscular dystrophy.
**Supplementary Figure 13**	Lm α2β1(G269R)γ1: class 1 mutation causing heart defects.
**Supplementary Figure 14**	Lm α5β2(Y48S)γ1: class 1 mutation leading to Pierson syndrome.
**Supplementary Figure 15**	Lm α5β2(S80R)γ1: class 1 mutation causing Pierson syndrome.
**Supplementary Figure 16**	Lm α5β2(H147R)γ1: class 1 mutation leading to Pierson syndrome.
**Supplementary Figure 17**	Lm α5β2(S179F)γ1: class 1 mutation causing Pierson syndrome.
**Supplementary Figure 18**	Lm α5β2(D167Y)γ1: class 2 mutations leading to Pierson syndrome.
**Supplementary Figure 19**	Lm α5β2(C321R)γ1: class 3 mutation leading to Pierson syndrome.
**Supplementary Figure 20**	Lm α2(Q167P)β1γ1: class 4 mutation causing limb-girdle muscular dystrophy.
**Supplementary Figure 21**	Lm α5β2(L139P)γ1: class 4 mutation leading to Pierson syndrome.
**Supplementary Discussion**	
**Supplementary References**	

Lm isoform	Sequence Length	Sequence Identity: LN/LN+LE1 [%]			
		α1 (*M.musculus*)	α1	α2	α5
**α1** (***M.musculus***)	249/305	---	90.76/90.49	77.11/74.43	52.94/53.74
**α1**	249/305	---	---	78.71/75.74	54.20/54.42
**α2**	249/305	---	---	---	53.36/52.72
**α5**	255/314	---	---	---	---
		**β1**	**β2**	**β3**	
**β1**	242/307	---	71.90/71.66	41.05/43.2	
**β2**	242/369	---	---	44.98/46.94	
**β3**	230/297	---	---	---	

Supplementary Table 1 Lm isoforms share significant sequence identity. The percent identity matrix for Lm α (α1, α2, α5) and β (β1, β2, β3) was calculated using Clustal Omega 1.2.4[1]. All sequences but one are from *Homo sapiens*. A sequence of Lm α1 from *Mus musculus* was included in the alignment, because the mouse protein was used for cryo-EM structure determination of Lm α1β1γ1[2]. The sequence identity is reported as a set of two numbers separated by a slash. The first number represents the protein sequence corresponding to the LN domain, whereas the second number represents the LN and LE1 domains. The length of sequences used for calculations is specified in the table. Please see Fig. 3 for more details.

Isoform	RMSD [Å]					
	Cryo-EM structure			X-ray structures		
**Cryo-EM structure**	**α1** (*Mus musculus*)	**β1**	**γ1**	**α5**	**β1**	**γ1**
**α1** (***M.musculus***)	---	5.49/4.93	3/3.27	2.61/2.74	4.51/4.61	3.39/3.3
**β1**	---	---	4.7/5.18	2.84/2.93	2.39/2.26	5.3/5.17
**γ1**	---	---	---	2.7/3.14	5.25/5.32	1.71/1.59

Supplementary Table 2 Lm isoforms are structurally homologous. Atomic models of α1, α5, β1 and γ1 from the cryo-EM[2] and X-ray structures[3,4] were superposed using UCSF ChimeraX[5] and UCSF Chimera[6]. The calculated RMSD values are reported as a set of two numbers separated by a slash. The first number represents the LN domain, whereas the second number represents the LN and LE1 domains. The RMSDs were calculated using all atoms present in the models (PDB ID: 8DMK[2]). The LN domains include the following residues: α1 (28-276), β1 (29-270), γ1 (37-283). Please see Fig. 4a for more details.

Class	Lm isoform	Altered residue	Analogous residue in Lmα1β1γ1	Phenotype	Structural defects*	Ref.
**1.** Binding interface	α1	Gln94Arg	Gln101	Poretti-Boltshauser syndrome	α1-γ1	[7]
	α2	Tyr138His	Tyr128	Laminin α2 Congenital muscular dystrophy	α2-β1	[8]
	α2	Ser204Phe	Ser194	Limb-girdle muscular dystrophy	α2-γ1	[9]
	α2	Ser277Leu	Ser267	Congenital muscular dystrophy	α2-γ1	[10]
	α2	Gly284Arg	Gly274	Limb-girdle muscular dystrophy	α2-β1	[11]
	α5	Arg286Leu	Arg263	Developmental disorder of kidney, face, and limbs	α5-γ1	[12]
	β1	Gly269Arg	Gly269	Heart defects	β1-γ1	[13]
	β2	Ser179Phe	Ala167	Pierson syndrome	β2-α5	[14]
	β2	Tyr48Ser	Tyr36	Pierson syndrome	β2-γ1	[15]
	β2	Ser80Arg	Ser68	Pierson syndrome	β2-α5	[16]
	β2	His147Arg	His135	Pierson syndrome	β2-γ1	[17]
**2.** Back face of β-sheet	α2	Ser157Phe	Ser147	Laminin α2 Congenital muscular dystrophy	α2	[18]
	β2	Asp167Tyr	Asp155	Pierson syndrome	β2	[19]
	β2	Arg246Gln	Arg234	Pierson syndrome	β2, may affect its N-glycosylation	[20]
	β2	Arg246Trp	Arg234	Pierson syndrome	β2, may affect its N-glycosylation	[21]
**3.** Disulfide bonds	α2	Cys83Arg	Cys73	Laminin α2 Congenital muscular dystrophy	α2, α2-γ1	[22]
	α2	Cys86Tyr	Cys76	Laminin α2 Congenital muscular dystrophy	α2, α2-γ1	[23]
	α2	Cys393Gly	Cys383	Limb-girdle muscular dystrophy	α2	[18]
	β2	Cys321Arg	Cys309	Pierson syndrome	β2, β2-γ1	[18]
**4.** Hydrophobic core	α2	Trp152Gly	Trp142	Limb-girdle muscular dystrophy	α2	[11]
	α2	Gln167Pro	Gln157	Limb-girdle muscular dystrophy	α2	[24]
	α2	Leu243Pro	Leu233	Limb-girdle muscular dystrophy	α2	[11]
	β2	Leu139Pro	Leu127	Pierson syndrome	β2	[16]
Non-polymerizing Lm	β3	Glu210Lys	---	Junctional epidermolysis bullosa		[25]
Mutations not observed in *Homo sapiens*	α1	Tyr258Cys	---	[*Mus musculus*] Eye defects		[26]
	β1	Glu198Lys	---	[*Drosophila melanogaster*] Heart defects		[13]
	β1	Ala209Glu	---	[*Drosophila melanogaster*] Heart defects		[13]

Supplementary Table 3 Pathogenic Lm mutations. The reported to date human pathogenic Lm mutations were categorized into four classes based on the underlying molecular defects they cause. Please see Fig. 4b for more details. In addition, one mutation detected in a non-polymerizing Lm β3, and three amino acid substitutions were identified in other species (not discussed in the current article). *The column lists affected Lm subunits and inter-subunit interfaces. Please see text for more details.

Lm polymer node	LN domain		LN-LE1 domains	
	RMSD [Å]	*No. atom pairs*	*RMSD [Å]*	*No. atom pairs*
**α1β1γ1**	0.82/1.56	228/249	0.94/2.16	272/305
α1(Q94R)β1γ1	0.82/1.47	228/249	0.92/2.12	271/305
**α2β1γ1**	0.81/1.32	228/249	0.99/1.53	260/307
α2(C83R)β1γ1	0.83/1.25	232/249	1.05/1.43	265/307
α2(C86Y)β1γ1	0.82/1.57	228/249	1.06/1.46	266/307
α2(Y138H)β1γ1	0.83/1.54	228/249	0.99/1.43	264/307
α2(W152G)β1γ1	0.82/1.53	228/249	1.1/1.49	264/307
α2(S157F)β1γ1	0.82/1.58	229/249	1.04/1.44	263/307
α2(Q167P)β1γ1	0.81/1.42	227/249	1/1.42	264/307
α2(S204F)β1γ1	0.83/1.56	229/249	0.99/1.79	226/307
α2(L243P)β1γ1	0.83/1.52	229/249	1.01/1.5	261/307
α2(S277L)β1γ1	0.84/1.18	232/249	1.07/1.57	244/307
α2(G284R)β1γ1	0.82/1.24	231/249	1.06/1.47	261/307
α2(C393G)β1γ1	0.82/1.58	228/249	0.98/1.47	259/307
α2β1(G269R)γ1	0.82/1.58	228/249	0.83/1.48	284/305
**α5β2γ1**	0.82/1.49	229/249	0.83/1.4	285/305
α5β2(Y48S)γ1	0.85/1.31	229/249	0.85/1.25	285/305
α5β2(S80R)γ1	0.86/1.28	232/249	0.86/1.21	288/305
α5β2(L139P)γ1	0.83/1.36	229/249	0.84/1.29	285/305
α5β2(H147R)γ1	0.83/1.37	229/249	0.84/1.29	285/305
α5β2(D167Y)γ1	0.83/1.38	228/249	0.85/1.31	284/305
α5β2(S179F)γ1	0.83/1.38	229/249	0.84/1.31	285/305
α5β2(R246Q)γ1	0.83/1.5	228/249	0.84/1.41	285/305
α5β2(R246W)γ1	0.83/1.47	228/249	0.85/1.38	285/305
α5β2(C321R)γ1	0.83/1.35	228/249	0.84/1.28	284/305
α5(R286L)β2γ1	0.83/1.42	229/249	0.85/1.35	285/305

Supplementary Table 4 Lm polymer nodes are structurally conserved. Twenty-six Lm polymer nodes, including three wild-type complexes (α1β1γ1, α2β1γ1, α5β2γ1), and twenty-three altered polymer nodes containing pathogenic mutations were modeled using AF2[27] and superposed with the cryo-EM structure of Lm α1β1γ1 (PDB ID: 8DMK[2]) using UCSF ChimeraX[5] and UCSF Chimera[6]. The calculated RMSD values for each LN and LN-LE1 domains are reported as a set of two numbers separated by a slash. The first number reflects values obtained for pruned atom pairs, whereas the second number represents pairwise-sequence alignment of all atoms. Predicted AF2 models reveal an extraordinary structure conservation among Lm polymer nodes, particularly in the regions representing hydrophobic cores located in the LN domains. The LN domains contain the following residues: α1 (28-276), β1 (29-270), γ1 (37-283). See Supplementary Fig. 7 for more details.

Lm polymer node	$\Delta\Delta G$ *stability [kcal/mol]*
**α1β1γ1**	
α1(Q101R)β1γ1	-0.85
**α2β1γ1**	
α2(C83R)β1γ1	-1.16
α2(C86Y)β1γ1	-1.09
α2(Y138H)β1γ1	-0.89
α2(W152G)β1γ1	-3.78
α2(S157F)β1γ1	-0.85
α2(Q167P)β1γ1	0.13
α2(S204F)β1γ1	-0.37
α2(L243P)β1γ1	-1.51
α2(S277L)β1γ1	-0.9
α2(G284R)β1γ1	-0.82
α2(C393G)β1γ1	0.15
α2β1(G269R)γ1	-0.8
**α5β2γ1**	
α5β2(Y48S)γ1	-1.68
α5β2(S80R)γ1	-0.43
α5β2(L139P)γ1	-1.4
α5β2(H147R)γ1	-0.85
α5β2(D167Y)γ1	-0.41
α5β2(S179F)γ1	-0.19
α5β2(R246Q)γ1	-0.79
α5β2(R246W)γ1	-0.52
α5β2(C321R)γ1	-1.13
α5(R286L)β2γ1	-0.55

Supplementary Table 5 Energetic effects of single point mutations on the stability of pathogenic Lm polymer nodes. We employed DynaMut2[28] to estimate energetic effects of twenty-three pathogenic mutations on the stability of Lm polymer nodes. The changes in ΔΔG [kcal/mol] resulting from individual single point mutations are listed in the table. Destabilizing effects of point mutations are reflected by the decrease in calculated ΔΔG values.

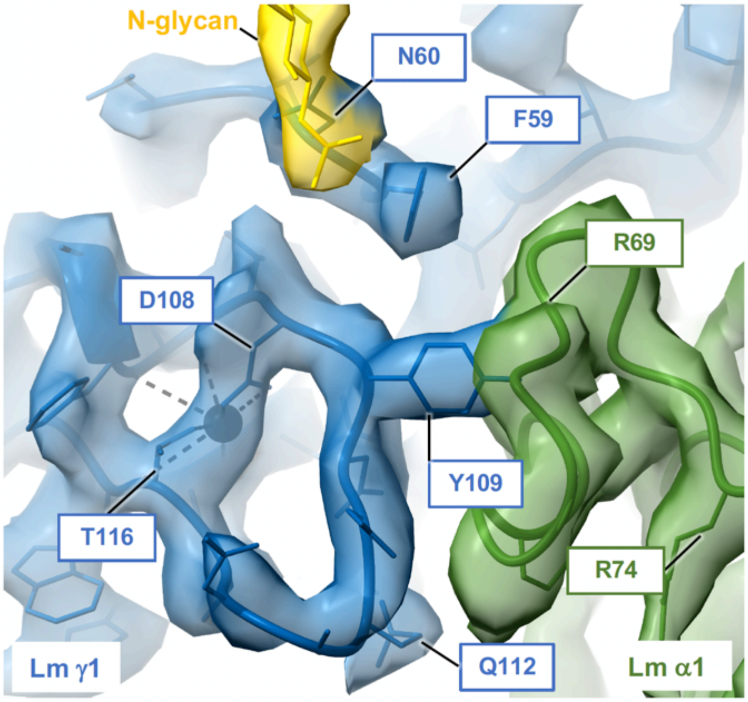

Supplementary Figure 1 Structural details of the α1-γ1 interface in Lm α1β1γ1 polymer node. Structural features of the loops stabilizing the α1-γ1 interface. Lm α1 and γ1 are shown in green and blue, respectively. The loop from γ1 contains residues coordinating a calcium ion (D108 and T116). The calcium ion is shown as a black sphere.

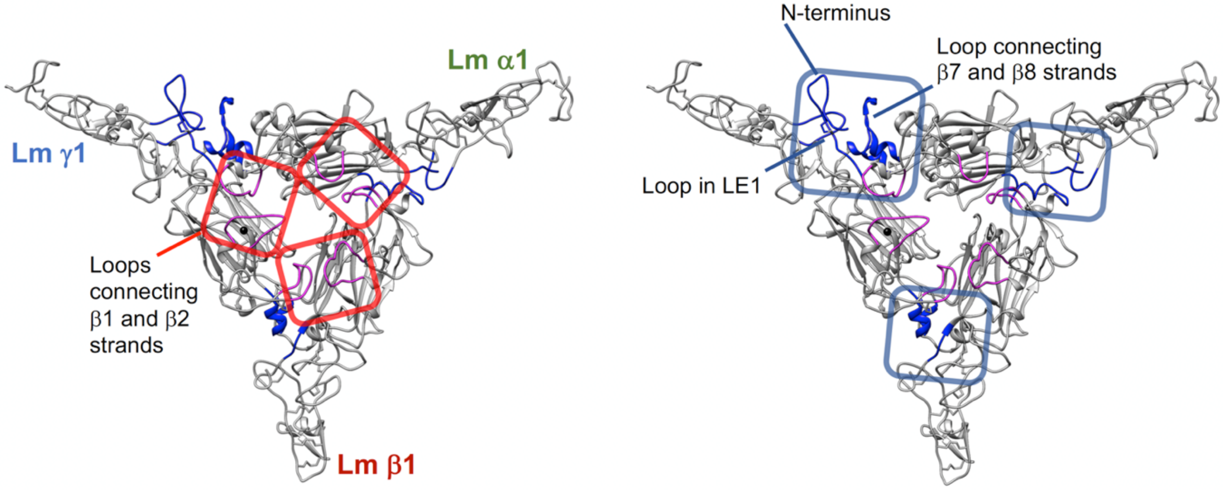

Supplementary Figure 2 Inter-subunit interactions stabilizing Lm α1β1γ1 polymer node. A structure of Lm node resembles two triangles positioned one on top of another, each representing a network of unique inter-subunit interactions. The first group involves loops connecting β1 and β2 strands in β-sheets from neighboring subunits within the trimeric complex. These regions are highlighted by red boxes. The second set of interactions highlighted by blue boxes involves loops linking strands β7 and β8 from one subunit and the N-terminal regions along with one of the loops from the LE1 domain of the neighboring subunit.

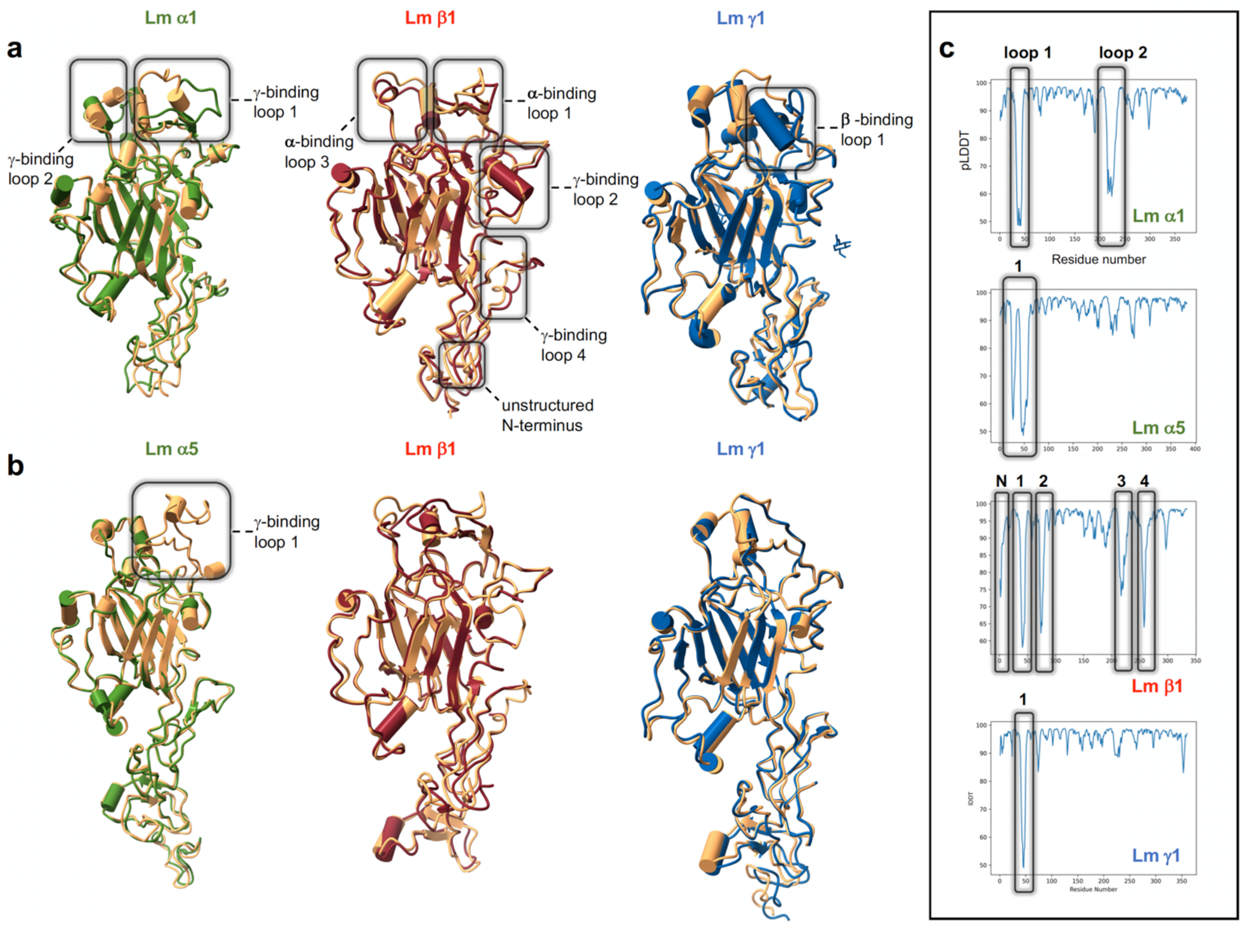

Supplementary Figure 3 Validation and superposition of monomeric AF2 models with the experimentally derived structures of Lm α1, α5, β1 and γ1. The predicted models are in excellent agreement with structures derived experimentally. Cryo-EM and X-ray structures are color-coded: α1 and α5 are shown in green, β1 is displayed in red, and γ1 is colored in blue. Corresponded AF2 models are shown in orange. (**a**) The backbone RMSDs calculated for the pairs of the AF2 model vs. a correspondent subunit from the cryo-EM structure of the trimeric Lm α1β1γ1[2] polymer node are: 2.97 Å across 305 residues for Lm α1 (PDB ID: 8DMK[2]), 1.58 Å for Lm β1 (306 residues, PDB ID: 8DMK[2]), 2 Å for Lm γ1 (303 residues, PDB ID: 8DMK[2]). **(b)** RMSDs calculated between crystal structures of α5, β1 and γ1, and correspondent AF2 models are: 1.12 Å for α5 (333 residues, PDB ID: 2Y38[4]), 1.93 Å for β1 (323 residues, PDB ID: 4AQS[3]), and 1.56 Å for γ1 (356 residues, PDB ID: 4AQT[3]). Please see Supplementary Table 3 for more details. (**c**) The accuracy of AF2 predictions is reflected by high pLDDT values with the baseline of approximately 98 for the modelled Lm chains. Some of the protein loops are modelled with less confidence, however the cryo-EM structure of Lm α1β1γ1[2] provides the guide allowing for validation of the local structures in these regions. These loop regions and correspondent pLDDT values at the pLDDT plots are highlighted in the figure by gray boxes. Importantly, only one out of twenty-three mutations underlying LN-lamininopathies described in this article is located in these regions, namely Ser 80 to arginine substitution in Lm α5β2(S80R) γ1. Ser 80/ Arg mutation has been previously extensively investigated structurally[2] and biochemically[29]. Furthermore, the AF2 model and the cryo-EM structure of Lm α1β1γ1[2] are in good agreement in this region. Please see Supplementary Figure 15 for details.

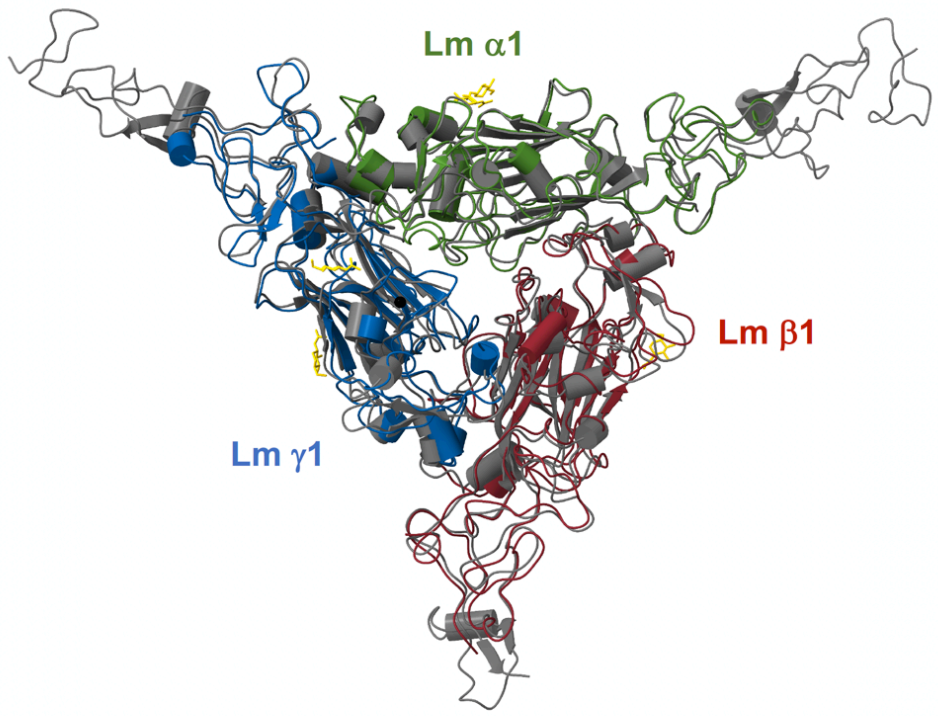

Supplementary Figure 4 Validation and superposition of the trimeric AF2 model with the cryo-EM structure of Lm α1β1γ1. Individual Lm chains from the cryo-EM structure[2] are color-coded, with α1 presented in green, β1 shown in red, and γ1 displayed in blue. The backbone RMSD across 305 atom pairs is 2.4 Å. Correspondent pLDDT values for the AF2 model are displayed in Supplementary Figure 8.

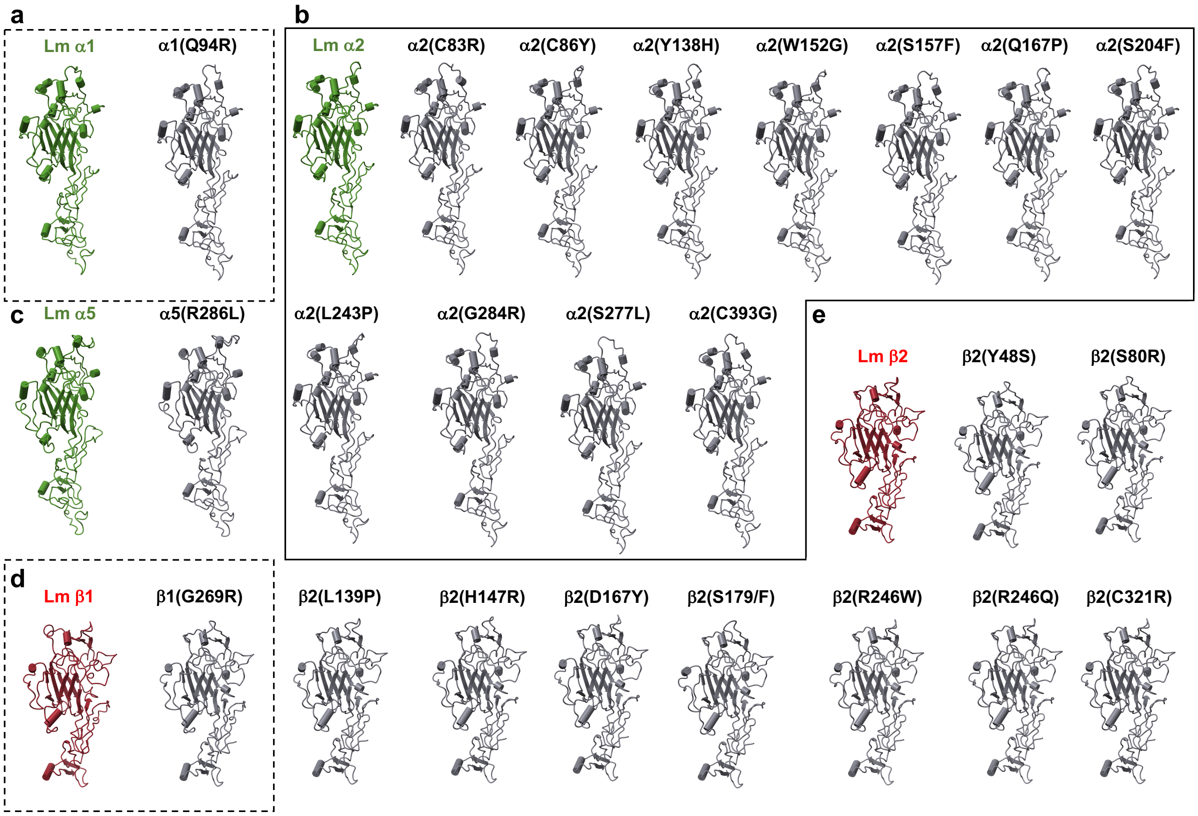

Supplementary Figure 5 AF2 models of wild-type Lm subunits and pathogenic Lm isoforms implicated in LN-lamininopathies. (**a**) Lm α1 and its mutant variant α1(Q94R). (**b**) Lm α2 and its altered isoforms (**c**) Lm α5 and a mutant isoform α5(R286L). (**d**) Lm β1and β1(G269R). (**e**) Lm β2 and its altered isoforms. The accuracy of AF2 predictions is reflected by high pLDDT values presented in Supplementary Figure 6 and also in Supplementary Figure 3c.

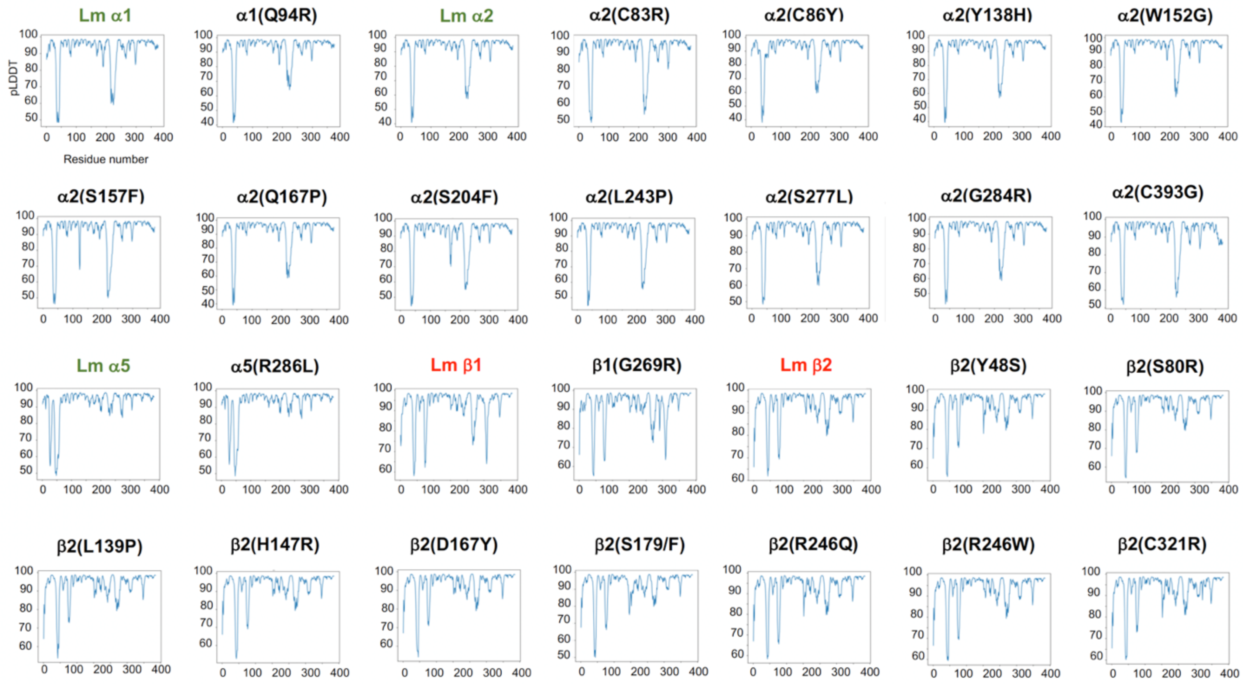

Supplementary Figure 6 The pLDDT plots calculated for the AF2 models of wild-type and pathogenic Lm isoforms implicated in LN-lamininopathies. The accuracy of AF2 predictions is reflected by high pLDDT values with the baseline of approximately 98 for all modelled Lm chains. Some of the loops in Lm subunits are predicted with lower confidence. These regions, containing only one of twenty-two pathogenic mutations (β2(S80R), Supplementary Figure 13), are in good agreement with the cryo-EM structure of Lm α1β1γ1[2]. The correspondent structural AF2 models are presented in Supplementary Figure 5.

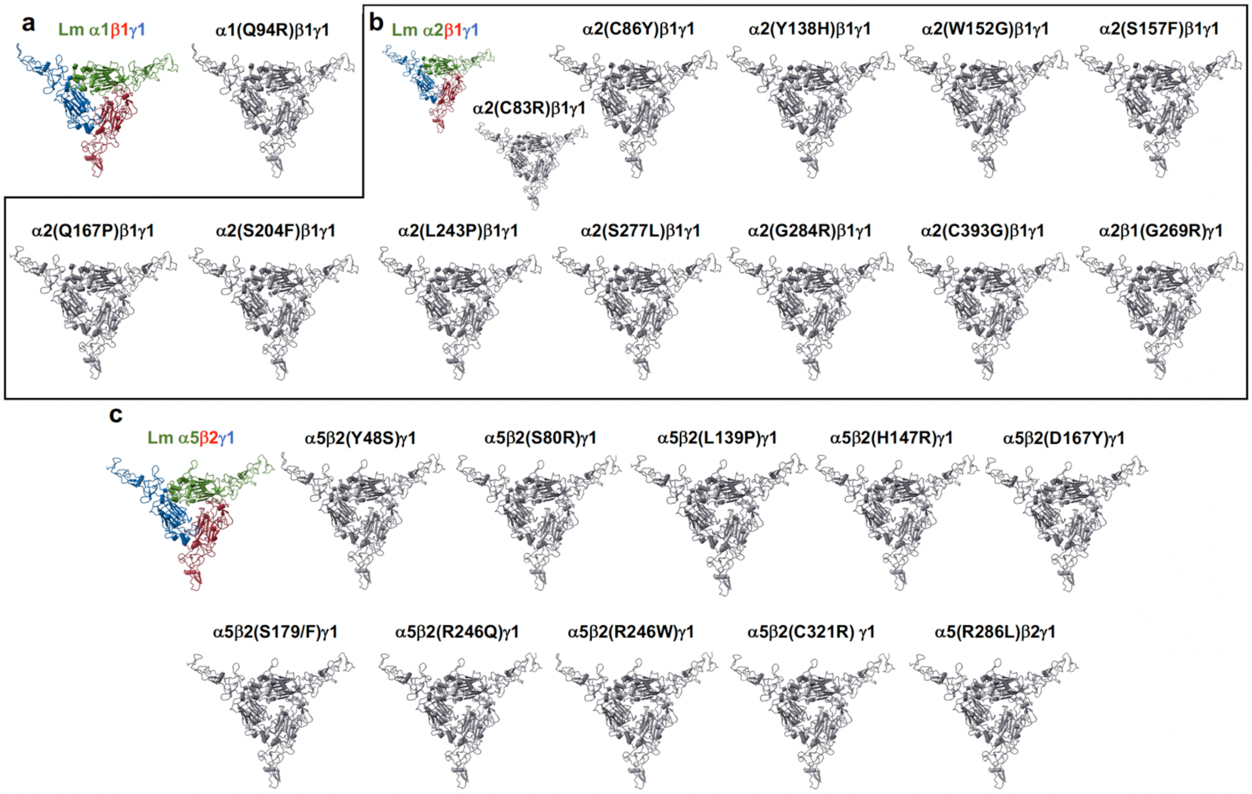

Supplementary Figure 7 AF2 models of trimeric wild-type Lm polymer nodes and their pathogenic counterparts causing LN-lamininopathies. (**a**) Lm α1β1γ1 and α1(Q94R)β1γ1. (**b**) Lm α2β1γ1 and its twelve altered variants (**c**) Lm α5β2γ1 and its ten pathogenic isoforms. The modeled structures are highly accurate as reflected by pLDDT values shown in Supplementary Figure 8.

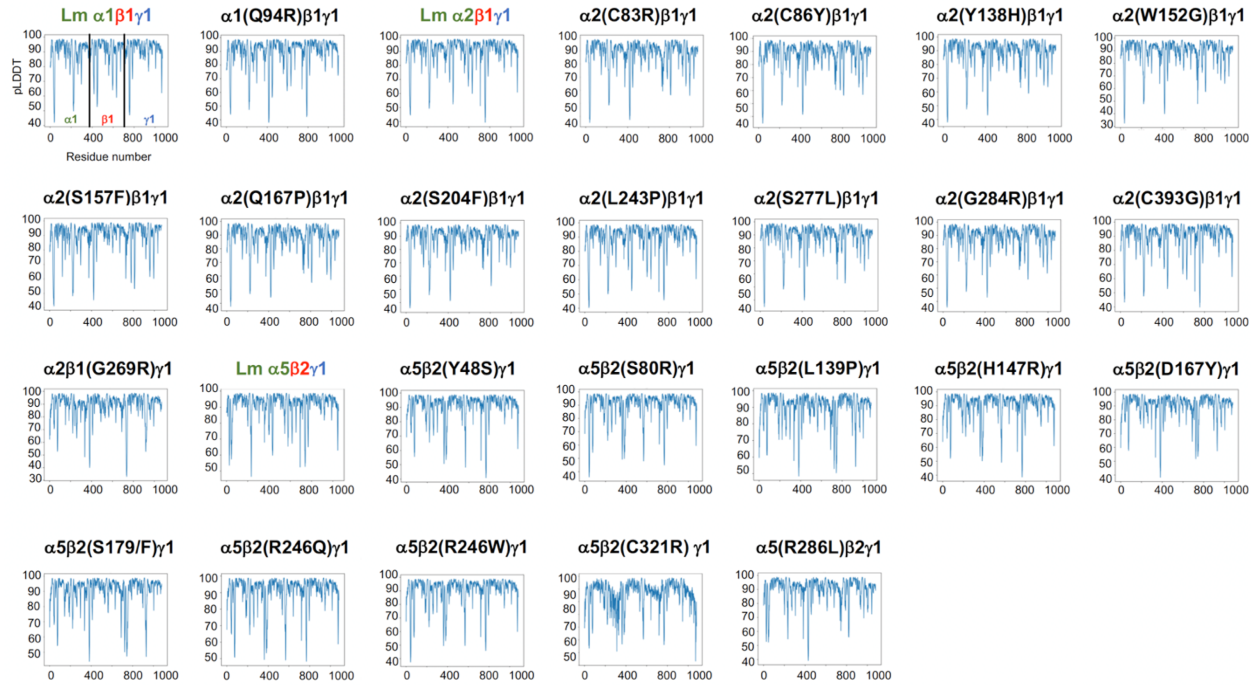

Supplementary Figure 8 The pLDDT plots calculated for the AF2 models of wild-type and pathogenic Lm polymer nodes. The accuracy of AF2 predictions is reflected by high pLDDT values with the baseline of approximately 98 for all modelled Lm polymer nodes. Some of the loops in Lm subunits are predicted with lower confidence. These regions contain only one of twenty-two pathogenic mutations underlying LN-lamininopathies (β2(S80R), Supplementary Figure 14, and are in good agreement with the cryo-EM structure of Lm α1β1γ1[2]. The correspondent structural AF2 models of Lm polymer nodes are presented in Supplementary Figure 7.

Lm α1(Q94R)β1γ1

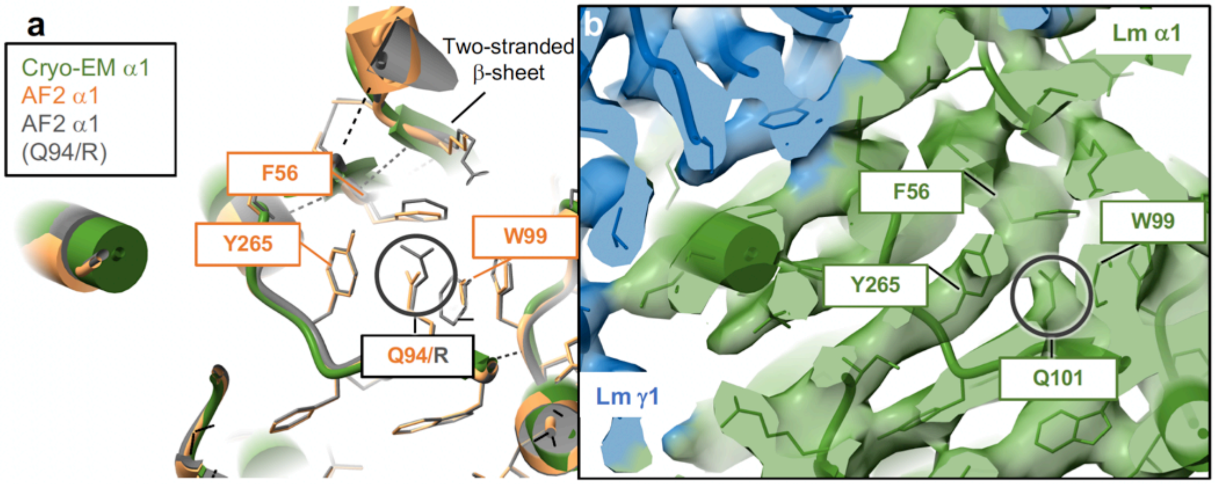

Supplementary Figure 9 Lm α1(Q94R)β1γ1: class 1 mutation causing Poretti-Boltshauser syndrome. (a) Mutation of Gln 101 to arginine in α1(Q94R)β1γ1 results in clashes of Arg 101 with neighboring α1 residues Phe 56 and Trp 99, destabilizing the α1-γ1 interaction. A substitution of Gln 94 with Arg in α1 leads to Poretti-Boltshauser syndrome[7]. **(b)** Side-chain conformations in the cryo-EM structure of Lm α1β1γ1[2] and in the AF2 model of α1(Q94R)β1γ1 are nearly identical in this region. The cryo-EM structure of Lm α1β1γ1, containing a mouse variant of Lm α1, revealed that Gln 101 interacts with residues from the inner face of the two-stranded β-sheet located in the toe region of α1. This β-sheet stabilizes the loop 1 at the α1-γ1 interface (Supplementary Figure 3a).

Lm α2(S204F)β1γ1

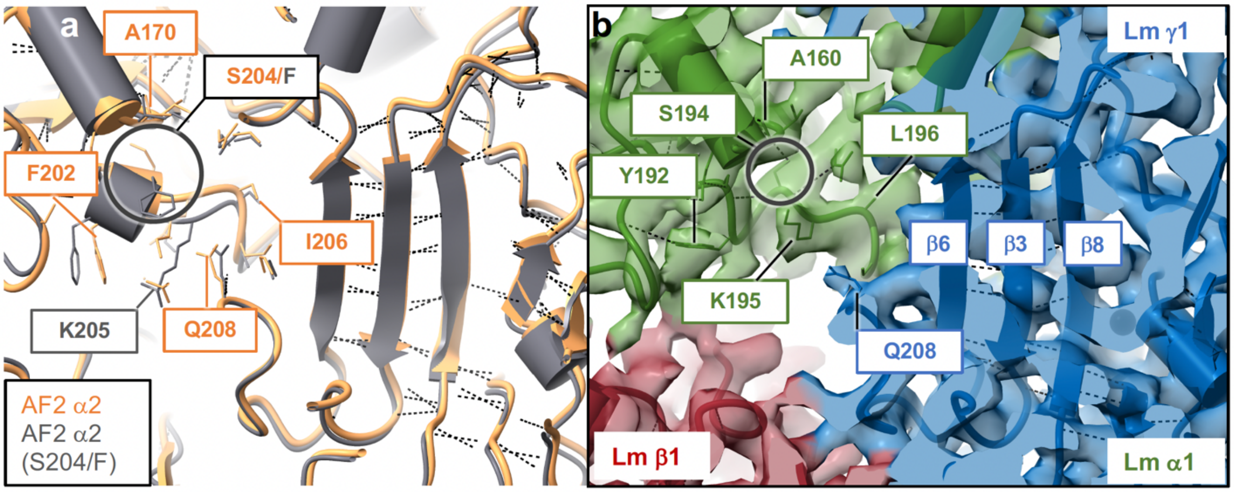

Supplementary Figure 10 Lm α2(S204F)β1γ1: class 1 mutation causing limb-girdle muscular dystrophy. (a) A mutation of Ser 204 in α2(S204F)β1γ1 to phenylalanine breaks a hydrogen bond formed by Ser 204 with Ala 170, which stabilizes the loop 1 forming the α2-γ1 interface (Supplementary Figure 3a). Consequently, the mutation disrupts Lm trimer assembly leading to LGMD[9]. **(b)** The cryo-EM structure of Lm α1β1γ1[2] revealed that Ser 194 and Ala 160, both from α1, form a hydrogen bond stabilizing analogous loop 1 at the α1-γ1 interface.

Lm α2(S277L)β1γ1

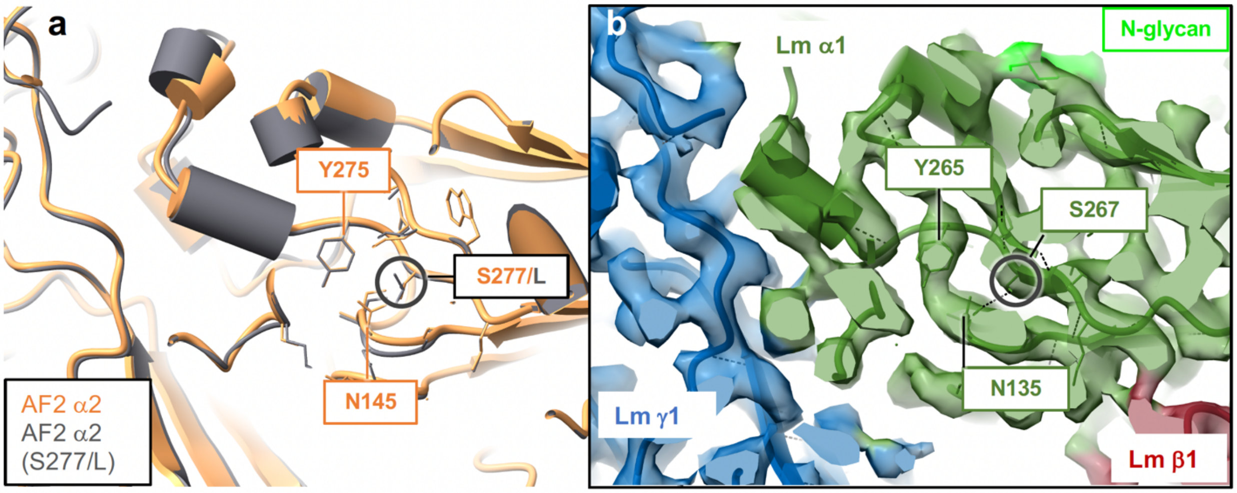

Supplementary Figure 11 Lm α2(S277L)β1γ1: class 1 mutation causing laminin α2 congenital muscular dystrophy. (a) Amino acid substitution of Ser 277 to leucine in α2(S277L)β1γ1 breaks a hydrogen bond formed by Ser 277 with its hydrogen donor Asn 145, while destabilizing the loop 1 (Supplementary Figure 3a) involved in formation of the α2-γ1 interface. The mutation leads to LAMA2-CMD[8]. **(b)** In a similar fashion, the cryo-EM structure of Lm α1β1γ1[2] revealed that Ser 267 from α1 forms a hydrogen bond with Asn 135, stabilizing the conformation of the loop 1 at the α1-γ1 interface.

Lm α2(G284R)β1γ1

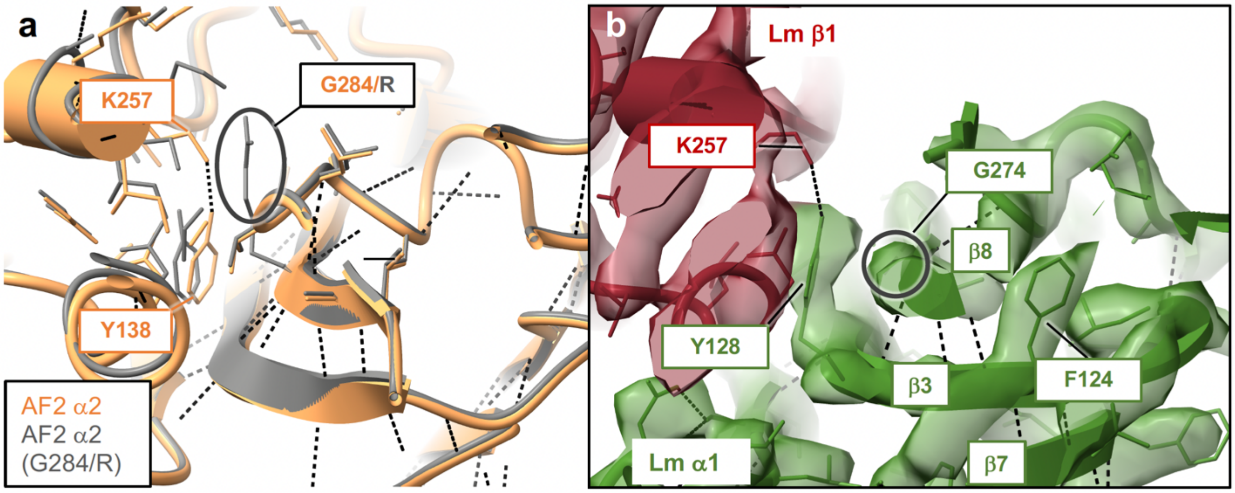

Supplementary Figure 12 Lm α2(G284R)β1γ1: class 1 mutation leading to limb-girdle muscular dystrophy. (a) A substitution of Gly 284 with arginine in α2(G284R)β1γ1destabilizes the α2-β1 interface. This defect is manifested as LGMD[9]. Large positively charged side chains of Arg 248 and Lys 257 repeal each other most likely breaking the hydrogen bond formed between Tyr 138 and Lys 257, subsequently destabilizing the α2-β1 interface. **(b)** In an analogous manner, Gly 274 from the cryo-EM structure of Lm α1β1γ1[2] is located in close proximity to Tyr 128 from α1 which, in turn, is involved in a hydrogen bonding interaction with β1's Lys 257. The hydrogen bond between the aforementioned residues stabilizes the α1-β1 interface.

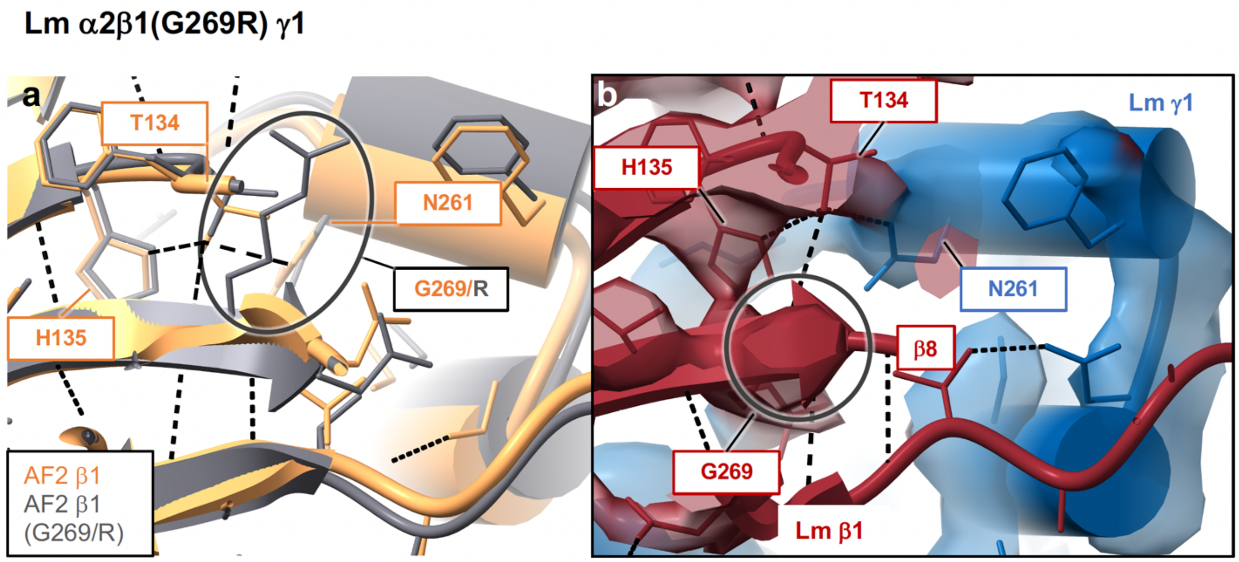

Supplementary Figure 13 Lm α2β1(G269R)γ1: class 1 mutation causing heart defects. (a) A mutation of Gly 269 to arginine in α2β1(G269R)γ1 disrupts a network of hydrogen bonds at the β1-γ1 interface leading to destabilization of the Lm trimer, resulting in heart defects[13] . A substitution of a small glycine's side chain with a large arginine's side chain directly affects a bond between Gly 269 from β1 and Thr 134 from β1, and indirectly disturbs another hydrogen bond involving Thr 134 and Asn 261 from γ1. **(b)** The cryo-EM structure of Lm α1β1γ1[2] reveals an identical network of hydrogen bonding interactions as described above.

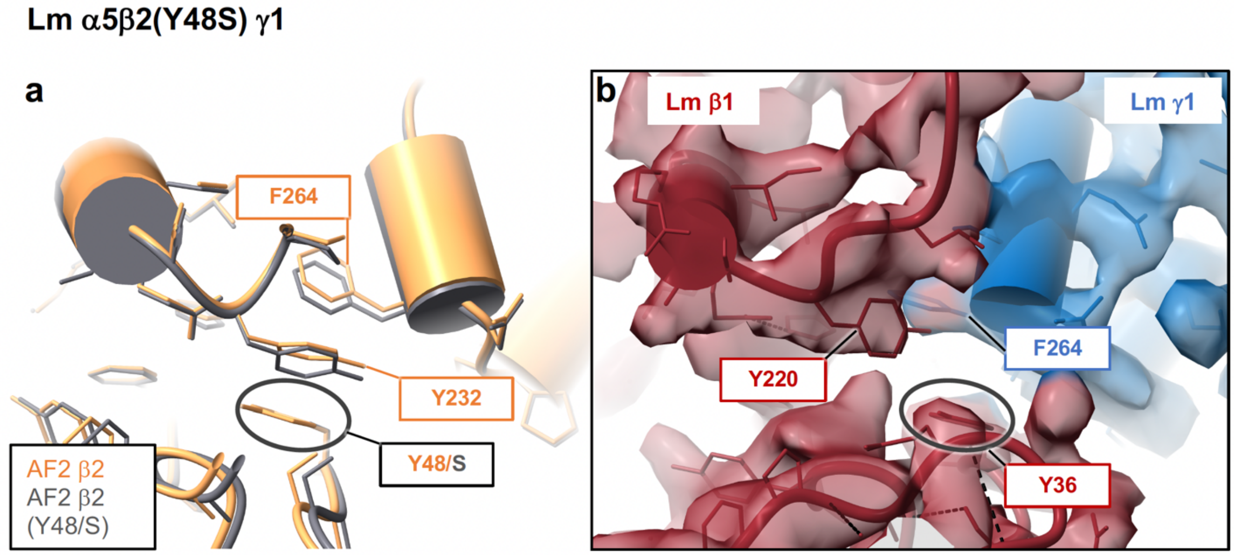

Supplementary Figure 14 Lm α5β2(Y48S)γ1: class 1 mutation leading to Pierson syndrome. (a) The replacement of Tyr 48 in α5β2(Y48S)γ1with polar serine breaks a network of hydrophobic stacking interactions stabilizing the β2-γ1 interface, subsequently leading to Pierson syndrome[15]. The interaction involves Tyr 48 and Tyr 230, both from β2, and Phe 264 from γ1. **(b)** An analogous network of interactions is formed by Tyr 36, Tyr 220, and Phe 264 in the cryo-EM structure of Lm α1β1γ1[2].

Lm α5β2(S80R) γ1

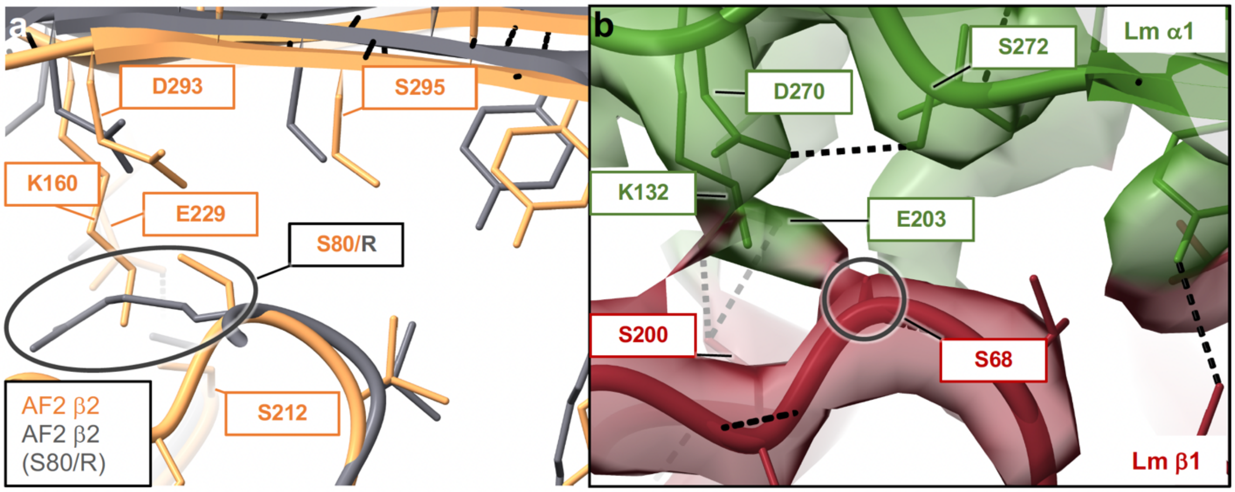

Supplementary Figure 15 Lm α5β2(S80R)γ1: class 1 mutation causing Pierson syndrome. (a) A substitution of Ser 80 into arginine in α5β2(S80R)γ1 leads to Pierson syndrome[16] by disturbing the α5-β2 interface. The Ser 80 to arginine mutation likely affects the hydrogen bonds formed by Ser 212 from β2 with Glu 229 from α5, and by Asp 293 with Ser 295, both from α5. **(b)** An analogous residue, Ser 68, from the cryo-EM structure of Lm α1β1γ1[2] is located in one of Lm β1 loops spanning amino acids I66-K75. This loop packs against the inner face of the β-sheet forming the α1-β1 interface. Previous SEC analysis of the Lm polymer node reconstituted with the wild-type α1 and γ1, and a mutant β1 harboring the Ser 68/ Arg mutation revealed that the altered Lm polymer node doesn't maintain its trimeric structure[29]. A substitution of neutral Ser 68 with a larger and positively charged arginine disrupts the network of neighboring hydrogen bonds stabilizing the β1-α1 interface, namely: a hydrogen bond between Asp 270 and Ser 272, both from α1, and most likely indirectly affects the inter-subunit hydrogen bond involving Ser 200 from β1 and Glu 203 from α1 through the electrostatic repealing of Lys 132 from α1 with an altered Arg 68.

Lm α5β2(H147R) γ1

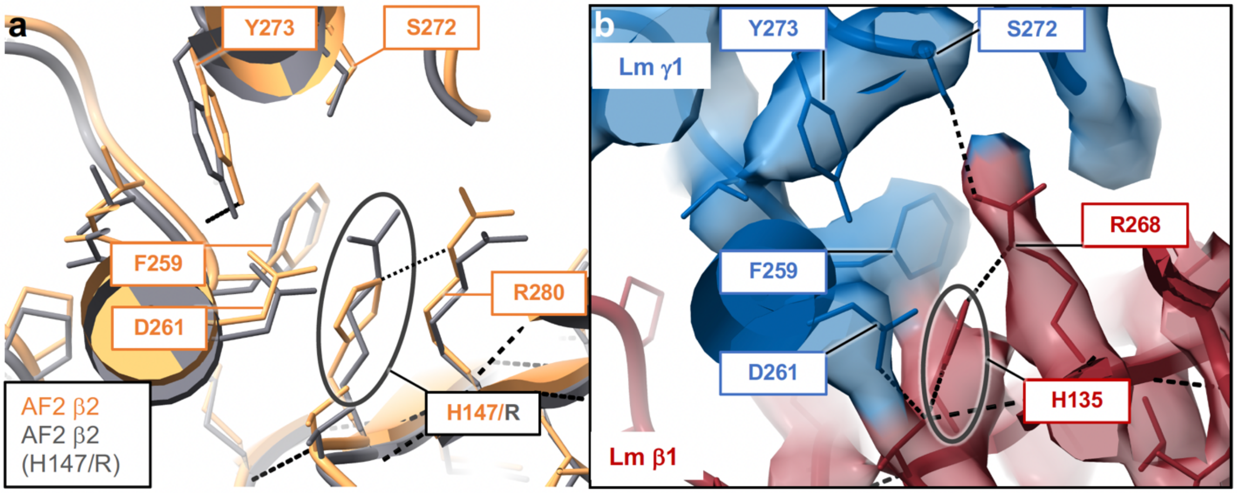

Supplementary Figure 16 Lm α5β2(H147R)γ1: class 1 mutation leading to Pierson syndrome. (a) Another example of Pierson syndrome-causing mutation[17] is a substitution of His 147 with arginine in Lm β2. The mutation directly disturbs a network of hydrogen bonds at the β2-γ1 interface in α5β2(H147R)γ1, namely the bonds between His 147 and Asp 261 from γ1, and His 147 and Arg 280 from β2. Another hydrogen bond involving Arg 280 from β2 and Ser 272 from γ1 is also indirectly affected. **(b)** The cryo-EM structure of Lm α1β1γ1[2] showed that analogous His 135 forms a similar network of hydrogen bonds stabilizing the β1-γ1 interface. In the cryo-EM structure His 135 directly interacts with Asp 261 from γ1, and with Arg 268 from β1. In turn, Arg 268 forms a hydrogen bond with Ser 272 from γ1.

Lm α5β2(S179/F)γ1

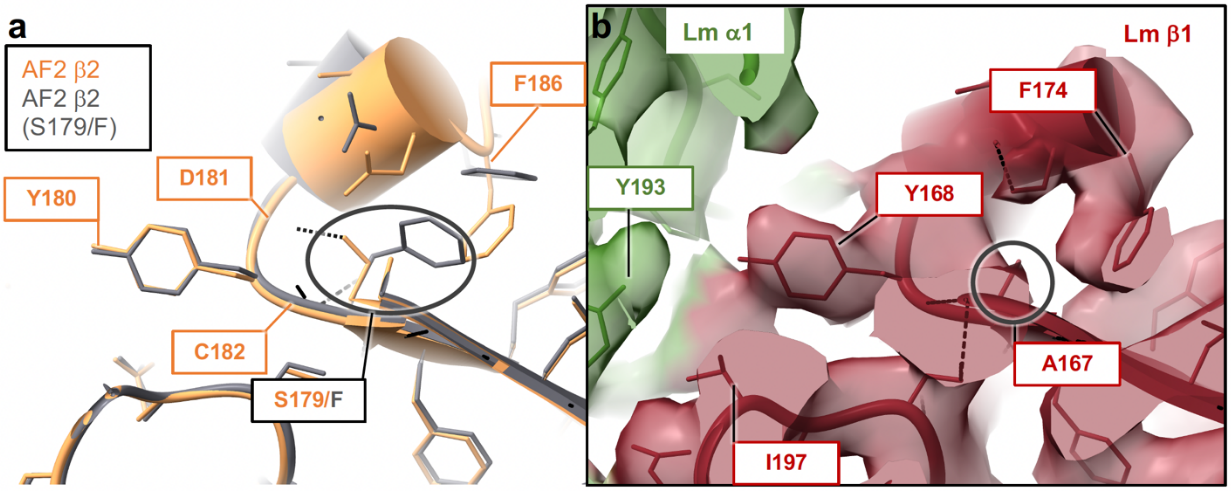

Supplementary Figure 17 Lm α5β2(S179F)γ1: class 1 mutation causing Pierson syndrome. (a) The replacement of Ser 179 with phenylalanine in α5β2(S179F)γ1 triggers Pierson syndrome[14]. Ser 179 stabilizes the conformation of a short loop from the β2-α5 interface. A mutation of Ser 179 to phenylalanine produces clashes with the neighboring Phe 186, which destabilize the loop and the β2-α5 interface. **(b)** In the cryo-EM structure of Lm α1β1γ1[2] Ala 167 is located in an analogous position as Ser 179. Its substitution with Phe would lead to clashes of Phe 167 with the neighboring Phe 174 from the loop involved in formation of the β1-α1 interface.

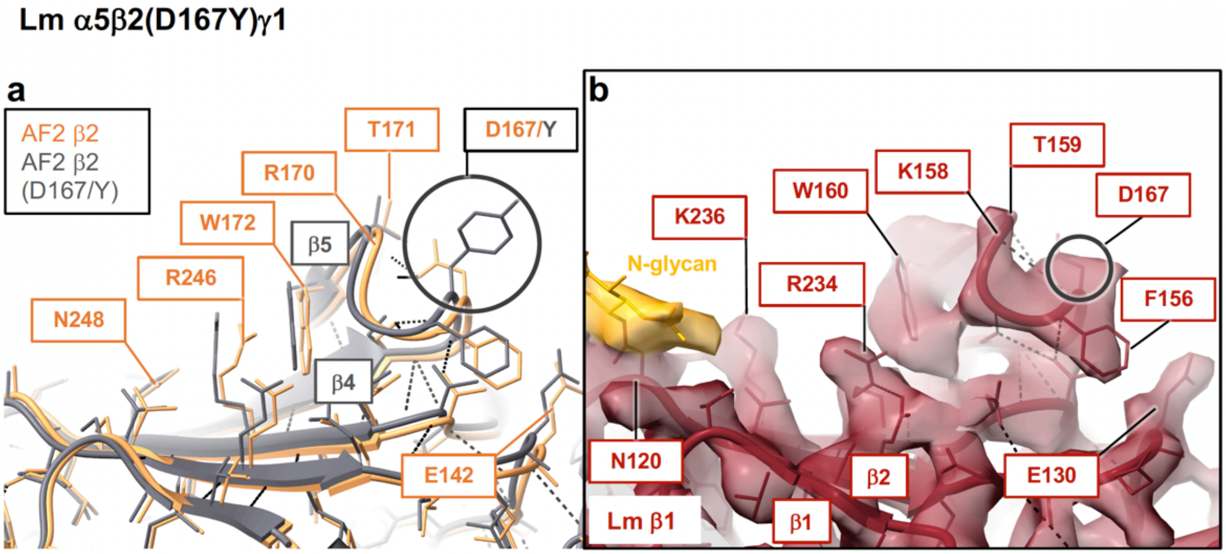

Supplementary Figure 18 Lm $\alpha5\beta2(D167Y)\gamma1$**: class 2 mutation leading to Pierson syndrome.** **(a)** The replacement of Asp 167 with tyrosine breaks the hydrogen bond formed by Asp 167 with the neighboring Arg 170, which in turn destabilizes a short loop connecting strands β4 and β5 at the back face of β2's jelly-roll motif. The Asp 167/ Tyr mutation in $\alpha5\beta2(D167Y)\gamma1$ leads to the development of Pierson syndrome[19-21]. **(b)** In a similar fashion, in the cryo-EM structure of Lm $\alpha1\beta1\gamma1$[2], Asp 167 from β1 forms hydrogen-bonds with Lys 158 and Thr 159 stabilizing the surface loop located in close proximity to the N-glycosylated Asn 120. AF2 models of wild-type and altered polymer nodes are displayed in orange and gray, respectively. The cryo-EM structure of Lm $\alpha1\beta1\gamma1$[2] is color-coded with α1, β1 and γ1 shown in green, red and blue, respectively.

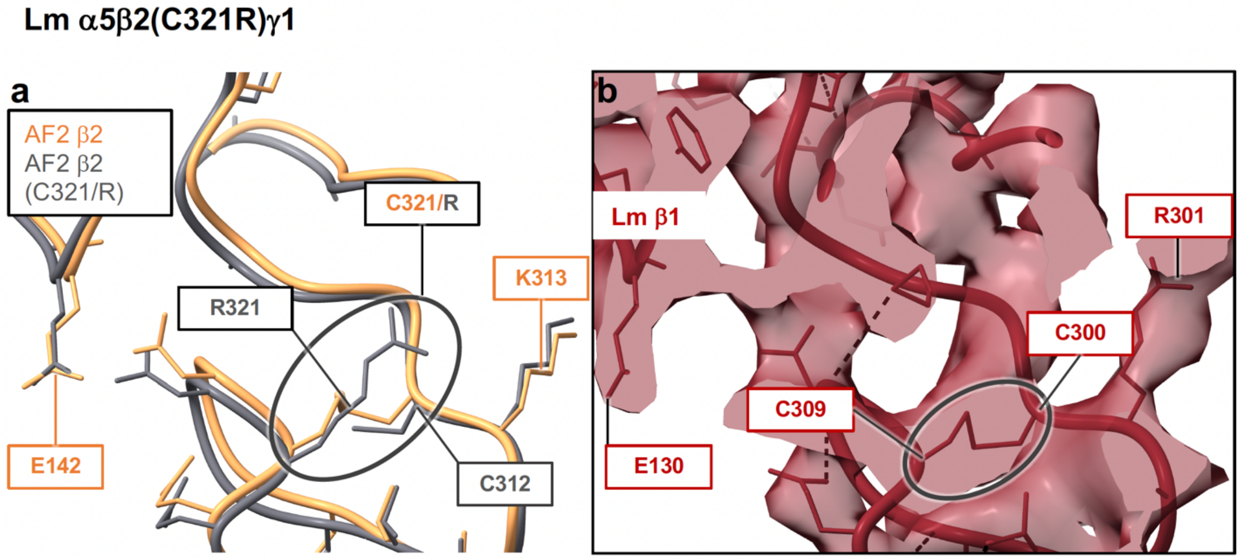

Supplementary Figure 19 Lm $\alpha5\beta2(C321R)\gamma1$**: class 3 mutation leading to Pierson syndrome. (a)** The Cys 321/Arg mutation, located in the LE1 domain of β2, leads to Pierson syndrome[30] by destabilizing the β2-γ1 interface. A substitution of Cys 321 with arginine breaks the disulfide bond formed by Cys 312 and Cys 321 involved in stabilization of LE1 domain structure and its interaction with γ1. **(b)** The cryo-EM structure of Lm $\alpha1\beta1\gamma1$[2] revealed an analogous disulfide bridge formed by Cys 300 and Cys 309 stabilizing the β1-γ1 interface.

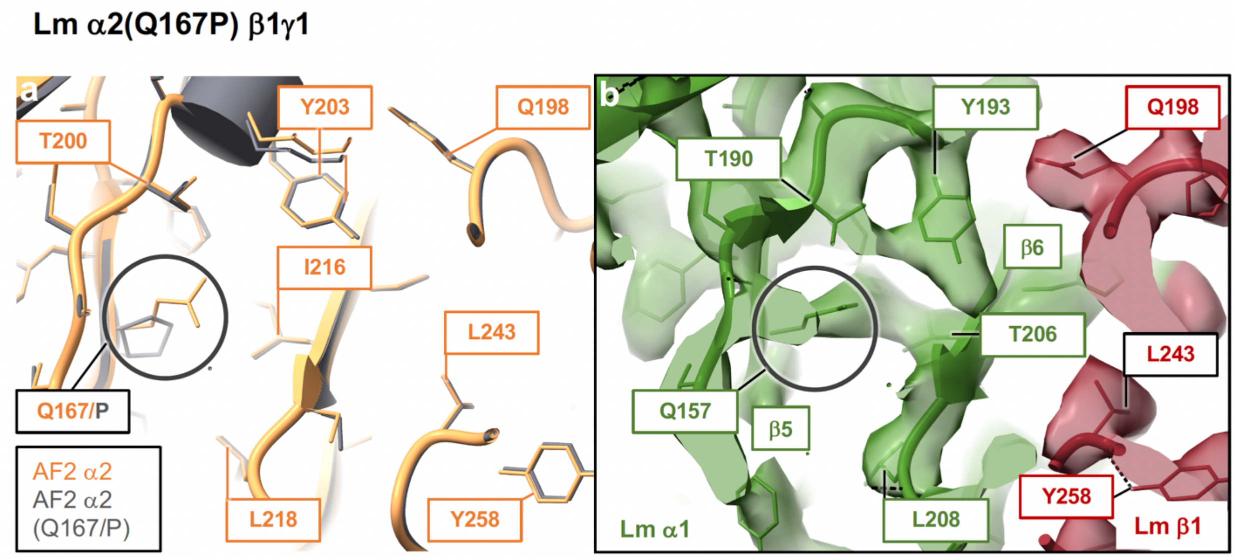

Supplementary Figure 20 Lm α2(Q167P)β1γ1: class 4 mutation causing limb-girdle muscular dystrophy. (a) A substitution of Gln 167 with proline in α2(Q167P)β1γ1 destabilizes the hydrophobic core of the jelly-roll motif in α2, and leads to LGMD[11,24]. The mutation affects folding of α2 subunit, and formation of the α2-β1 binding interface. **(b)** The cryo-EM structure of Lm α1β1γ1[2] revealed that an analogous Gln 157 is involved in stabilization of the hydrophobic core of α1's jelly-roll motif, thus it affects formation of the inter-subunit interface between α1 and β1.

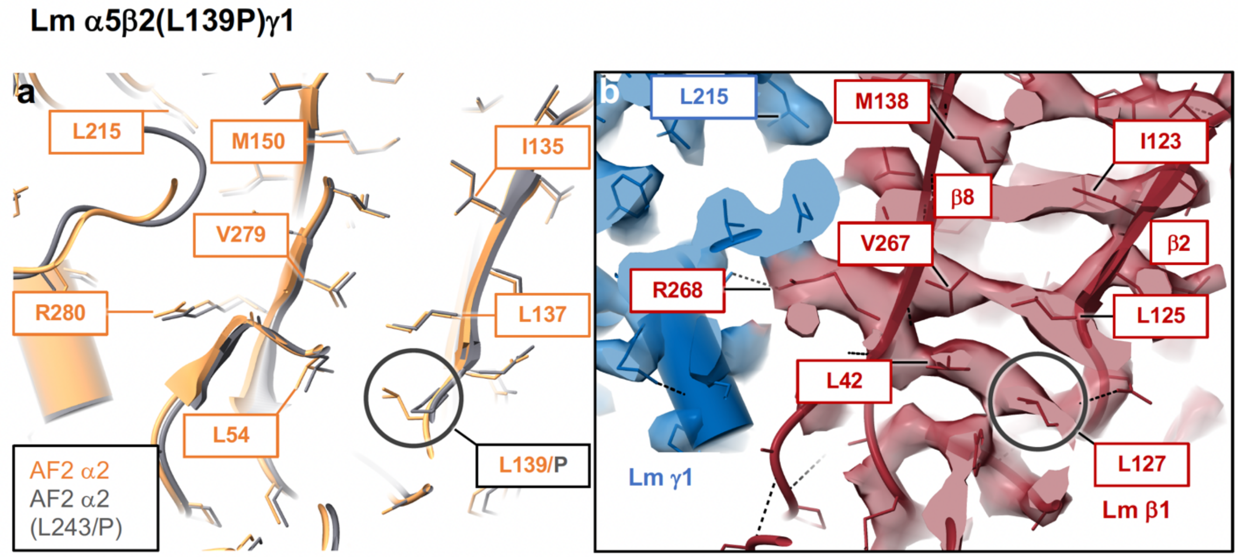

Supplementary Figure 21 Lm α5β2(L139P)γ1: class 4 mutation leading to Pierson syndrome. (a) The replacement of Leu 139 with proline in β2 subunit affects a network of hydrophobic interactions involving Leu 139, Leu 54, Leu 137, and Val 279, which stabilize the structure of β2's jelly-roll motif. The mutation likely interferes with folding of β2 and formation of the β2-γ1 interface in α5β2(L139P)γ1, subsequently leading to the development of Pierson syndrome[16]. **(b)** The cryo-EM structure of Lm α1β1γ1[2] displayed an identical network of interactions between Leu 127, Leu 42, Leu 125 and Val 267, which collectively stabilize the hydrophobic core of β1, and the β1-γ1 interface.

Supplementary Discussion

Mutations affecting the inter-subunit binding interfaces in Lm polymer nodes (class 1). A substitution of Gln 94 with Arg in α1 causes Poretti-Boltshauser syndrome[7]. The cryo-EM structure of Lm α1β1γ1[2] revealed that analogous Gln 101 interacts with residues from the inner face of the two-stranded β-sheet located in the toe region of α1 (**Supplementary Figure 9b**). This β-sheet, in turn, stabilizes the loop 1 at the α1-γ1 interface (**Supplementary Figure 3a)**. Side-chain conformations in the cryo-EM structure (**Supplementary Figure 9b**) and in the AF2 model (**Supplementary Figure 9a**) of α1(Q94R)β1γ1 are identical in this region. Mutation of Gln 94 to arginine in α1(Q94R)β1γ1 results in clashes of Arg 94 with neighboring α1 residues Phe 56 and Trp 99, destabilizing the α1-γ1 interaction (**Supplementary Figure 9a**). Another mutation of Ser 204 to phenylalanine in α2(S204F)β1γ1 is a causative factor of LGMD[9]. The cryo-EM structure revealed that Ser 194 and Ala 160, both from α1, form a hydrogen bond stabilizing the loop 1 at the α1-γ1 interface (**Supplementary Figure 10b**). Mutation of homologous Ser 204 in α2(S204F)β1γ1 to phenylalanine breaks the hydrogen bond with α2 Ala 170, subsequently affecting formation of the α2-γ1 interface (**Supplementary Figure 10a**). In a similar fashion, the cryo-EM structure revealed that Ser 267 from α1 is involved in a hydrogen-bonding interaction with Asn 135, also stabilizing the conformation of the loop 1 at the α1-γ1 interface (**Supplementary Figure 11b).** The mutation of analogous Ser 277 to leucine in α2(S277L)β1γ1, breaks a hydrogen bond formed by Ser 277 with its hydrogen donor, Asn 145 (**Supplementary Figure 11a)**, affecting Lm oligomerization and leading to LAMA2-CMD[8]. Another example is the disruption of the α2-β1 interface by a substitution of Gly 284 with arginine in α2(G284R)β1γ1 (**Supplementary Figure 12a)**. Large positively-charged side chains of Arg 248 and Lys 257 (equivalents of Gly 274 and Lys 257 in the cryo-EM structure, **Supplementary Figure 12b**) repeal each other likely breaking the hydrogen bond formed between Tyr 138 and Lys 257, which role is to stabilize the α2-β1 interface (**Supplementary Figure 12a)**. This molecular defect is manifested as LGMD[9]. The Gly 269 mutation to arginine in α2β1(G269R)γ1 leads to heart defects[13] by disrupting a network of hydrogen bonds at the β1-γ1 interface. Both, the AF2 model (**Supplementary Figure 13a)** and the cryo-EM structure (**Supplementary Figure 13b)** revealed that Gly 269 forms a hydrogen bond, with Thr 134 which, in turn, is involved in hydrogen-binding interaction with Asn 261 from γ1. A substitution of Gly 269 with a large arginine side chain breaks the latter interaction while destabilizing the inter-subunit interface. In Pierson syndrome, the replacement of Tyr 48 with polar serine in α5β2(Y48S)γ1[15] breaks a network of hydrophobic stacking interactions stabilizing the β2-γ1 interface, which involves Tyr 48 and Tyr 230 from β2, and Phe 264 from γ1 (**Supplementary Figure 14a)**. Analogous network of interactions is formed by Tyr 36, Tyr 220, and Phe 264 in the cryo-EM structure of Lm α1β1γ1[2] (**Supplementary Figure 14b).** A substitution of Ser 80 into arginine in α5β2(S80R)γ1 also leads to Pierson syndrome[16] (**Supplementary Figure 15)**. Previous SEC analysis of the Lm complexes reconstituted with the wild-type α1 and γ1, and a mutant β1 harboring analogous Ser 68/Arg mutation, revealed that the trimeric Lm polymer nodes are not formed in the presence of the altered protein[29]. Ser 68 is located in one of Lm β1 loops spanning residues I66-K75. This loop packs against the inner face of the β-sheet in α1. A substitution of neutral Ser 68 with a larger and positively charged arginine disrupts the network of neighboring hydrogen bonds stabilizing the β1-α1 interface, including a hydrogen bond between Asp 270 and Ser 272, both in α1. The mutation most likely also indirectly affects the inter-subunit hydrogen bond involving Ser 200 in β1 and Glu 203 in α1 through the electrostatic repealing of Lys 132 from α1 with an altered Arg 68. (**Supplementary Figure 15b)**. Likewise, the Ser 80 to arginine substitution in α5β2(H147R)γ1 affects the hydrogen bonds formed by Ser 212 with Glu 229, and Asp 293 with Ser 295 (**Supplementary Figure 15a)**. Another Pierson syndrome-causing mutation[17] is a substitution of His 147 into arginine. The cryo-EM structure showed that analogous His 135 forms a network of hydrogen bonds stabilizing the β1-γ1 interface. In the cryo-EM structure, His 135 directly interacts with Asp 261 from γ1, and with Arg 268 from β. The latter residue, in turn, forms a hydrogen bond with Ser 272

(**Supplementary Figure 16b**). In a similar fashion, hydrogen bonds involving the following residues His 147-Asp 261, His 147-Arg 280, and Arg 280-Ser 272 are affected by the mutation of Ser 80 to arginine, destabilizing the β2-γ1 interface in α5β2(H147R)γ1 (**Supplementary Figure 16a)**. The replacement of Ser 179 with phenylalanine in α5β2(S179F)γ1 also triggers Pierson syndrome[14]. At the molecular level Ser 179 stabilizes conformation of a short loop from the β2-α5 interface (**Supplementary Figure 17a)**. A mutation of Ser 179 to phenylalanine produces clashes with the neighboring Phe 186 (equivalent to Phe 174 in the cryo-EM structure, **Supplementary Figure 17b**), which leads to destabilization of the loop at the inter-subunit β2-α5 interface.

Mutations located in close proximity to the N-glycosylation sites on the back face of the jelly-roll motifs in Lm subunits (class 2). Analogously to α2(S157F) mutation in α2(S157F)β1γ1 described in the main article, the replacement of Asp 167 with tyrosine in α5β2(D167Y)γ1 destabilizes a short loop containing residues Ser 167-Trp 172 in β2 (**Supplementary Table 5**). This loop connects strands β4 and β5 at the back face of the jelly-roll motif (**Supplementary Figure 18a)**. The β2(D167Y) mutation manifests in Pierson syndrome[14]. In a similar fashion, in the cryo-EM structure, Asp 167 from β1 is involved in hydrogen-bonding with Lys 158 and Thr 159 (**Supplementary Figure 18b)**. Both interactions stabilize the short loop consisting of residues Arg 152-Gly 161.

Mutations disrupting formation of disulfide bonds in Lm subunits (class 3). The Cys 321/Arg mutation, located in the LE1 domain of β2, has been implicated in Pierson syndrome[25]. A substitution of Cys 321 with arginine breaks the disulfide bond formed by Cys 321 with its binding partner Cys 312 (**Supplementary Figure 19a**). Because the LE1 domain is involved in binding to γ1 (**Supplementary Figure 2**), the Cys 321/Arg mutation destabilizes the β2-γ1 interface. The cryo-EM structure revealed analogous disulfide bridge formed by Cys 309 and Cys 300 (**Supplementary Figure 19b**). The Cys 393/Gly mutation from the LE2 domain of α2 triggers LGMD[31] (**Supplementary Table 3**). Although the density representing the LE2 is present in the cryo-EM Coulomb map of Lm α1β1γ1[2] (**Fig. 2a**), the intrinsic planar and rotational flexibility of LE rods revealed by the principal components analysis of the cryo-EM data[2] impedes molecular model building in this region. Thus, we decided not to interpret the significance of the Cys 393/Gly mutation, as we would not be able to verify it by comparing with the experimentally-derived structure. We speculate that the Cys 393/ Gly mutation affects the folding of the LE2 domain of α2, possibly disrupting the interaction between the neighboring Lm polymer nodes, and thus formation of the Lm lattice. Interestingly, the Cys 393/Gly mutation marginally increases the stability of Lm α2(C393G)β1γ1, as evidenced by the change in calculated ΔΔG (**Supplementary Table 5**).

Mutations affecting hydrophobic cores of Lm subunits (class 4). A substitution of Gln 167 with proline in α2(Q167P)β1γ1 affects the stability of α2's jelly-roll motif (**Supplementary Figure 20**). Interestingly, unlike 21/23 investigated mutations, the Gln 167/Pro alternation marginally increases the stability of Lm α2(Q167P)β1γ1, as evidenced by the change in calculated ΔΔG (**Supplementary Table 5**). In contrast, Leu 139/ Pro mutation occurs in β2 subunit, and it has a destabilizing effect on α5β2(L139P)γ1 (**Supplementary Table 5**). The above results indicate that both negative and positive changes in ΔΔG may affect formation of functional Lm polymer nodes, possible by indirectly altering the strength of the inter-subunit interactions. The replacement of leucine with proline affects a network of hydrophobic interactions involving Leu 54, Leu 139 and Val 279, which stabilize the structure of β2's jelly-roll motif (**Supplementary Figure 21**). The Leu 139/ Pro mutation may interfere with β2 folding and formation of the β2-γ1 interface in α5β2(L139P)γ1, subsequently leading to the development of Pierson syndrome[16].

Supplementary References

1. Sievers, F. et al. Fast, scalable generation of high-quality protein multiple sequence alignments using Clustal Omega. *Mol. Syst. Biol.* **7**, 539 (2011).
2. Kulczyk, A.W. et al. Cryo-EM reveals the molecular basis of laminin polymerization and LN-lamininopathies. *Nat. Commun.* **14**, 317 (2023).
3. Carafoli, F., Hussain, S.A. & Hohenester, E. Crystal structures of the network-forming short-arm tips of the laminin beta1 and gamma1 chains. *PLoS One* **7**, e42473 (2012).
4. Hussain, S.A., Carafoli, F. & Hohenester, E. Determinants of laminin polymerization revealed by the structure of the alpha5 chain amino-terminal region. *EMBO Rep.* **12**, 276-82 (2011).
5. Pettersen, E.F. et al. UCSF ChimeraX: Structure visualization for researchers, educators, and developers. *Protein Sci.* **30**, 70-82 (2021).
6. Pettersen, E.F. et al. UCSF Chimera-a visualization system for exploratory research and analysis. *J. Comput. Chem.* **25**, 1605-12 (2004).
7. Powell, L. et al. Identification of LAMA1 mutations ends diagnostic odyssey and has prognostic implications for patients with presumed Joubert syndrome. *Brain Commun.* **3**, 3(3):fcab163 (2021).
8. Oliveira, J. et al. LAMA2 gene analysis in a cohort of 26 congenital muscular dystrophy patients. *Clin. Genet.* **74**, 502-12 (2008).
9. Harris, E. et al. Clinical and neuroimaging findings in two brothers with limb girdle muscular dystrophy due to LAMA2 mutations. *Neuromuscul Disord.* **27**, 170-174 (2017).
10. Beytia Mde, L. et al. High creatine kinase levels and white matter changes: clinical and genetic spectrum of congenital muscular dystrophies with laminin alpha-2 deficiency. *Mol. Cell. Probes* **28**, 118-22 (2014).
11. Gavassini, B.F. et al. Clinical and molecular characterization of limb-girdle muscular dystrophy due to LAMA2 mutations. *Muscle Nerve* **44**, 703-9 (2011).
12. Jones, L.K. et al. A mutation affecting laminin alpha 5 polymerisation gives rise to a syndromic developmental disorder. *Development* **147**(21):dev189183 (2020).
13. Hollfelder, D., Frasch, M. & Reim, I. Distinct functions of the laminin β LN domain and collagen IV during cardiac extracellular matrix formation and stabilization of alary muscle attachments revealed by EMS mutagenesis in Drosophilla. *BMC Dev. Biol.* **14**, 24-26 (2014).
14. Choi, H.J. et al. Variable phenotype of Pierson syndrome. *Pediatr Nephrol* **23**, 995-1000 (2008).
15. Schapiro, D. et al. Panel sequencing distinguishes monogenic forms of nephritis from nephrosis in children. *Nephrol. Dial. Transplant.* **34**, 474-485 (2019).
16. Matejas, V. et al. Mutations in the human laminin beta2 (LAMB2) gene and the associated phenotypic spectrum. *Hum. Mutat.* **31**, 992-1002 (2010).
17. Mohney, B.G. et al. A novel mutation of LAMB2 in a multigenerational mennonite family reveals a new phenotypic variant of Pierson syndrome. *Ophthalmology* **118**, 1137-44 (2011).
18. Geranmayeh, F. et al. Genotype-phenotype correlation in a large population of muscular dystrophy patients with LAMA2 mutations. *Neuromuscul. Disord.* **20**, 241-50 (2010).
19. Kagan, M., Cohen, A.H., Matejas, V., Vlangos, C. & Zenker, M. A milder variant of Pierson syndrome. *Pediatr. Nephrol.* **23**, 323-7 (2008).
20. Zenker, M. et al. Human laminin beta2 deficiency causes congenital nephrosis with mesangial sclerosis and distinct eye abnormalities. *Hum. Mol. Genet.* **13**, 2625-32 (2004).
21. Hasselbacher, K. et al. Recessive missense mutations in LAMB2 expand the clinical spectrum of LAMB2-associated disorders. *Kidney. Int.* **70**, 1008-12 (2006).
22. Patton, B.L., Wang, B., Tarumi, Y.S., Seburn, K.L. & Burgess, R.W. A single point mutation in the LN domain of LAMA2 causes muscular dystrophy and peripheral amyelination. *J. Cell. Sci.* **121**, 1593-604 (2008).
23. Oliveira, J. et al. LAMA2 gene mutation update: Toward a more comprehensive picture of the laminin-alpha2 variome and its related phenotypes. *Hum. Mutat.* **39**, 1314-1337 (2018).

24. Rajakulendran, S., Parton, M., Holton, J.L. & Hanna, M.G. Clinical and pathological heterogeneity in late-onset partial merosin deficiency. *Muscle. Nerve.* **44**, 590-3 (2011).
25. Chen, Y.M. et al. Laminin beta2 gene missense mutation produces endoplasmic reticulum stress in podocytes. *J. Am. Soc. Nephrol.* **24**, 1223-33 (2013).
26. Di Blasi, C. et al. LAMA2 Gene Analysis in Congenital Muscular Dystrophy New Mutations, Prenatal Diagnosis, and Founder Effect. *Arch. Neurol..* **62**, 1582-1586 (2005).
27. Mellerio, J.E., Eady, R.A.J., Atherton, D.J., Lake, B.D. & McGrath, J.A. E210K mutation in the gene encoding the β3 chain of laminin-5 (LAMB3) is predictive of a phenotype of generalized atrophic benign epidermolysis bullosa. *Br. J. of Dermatol.* **139**, 325-331 (1998).
28. Edwards, M.M. et al. Mutations in Lama1 disrupt retinal vascular development and inner limiting membrane formation. *J. Biol. Chem.* **285**, 7697-711 (2010).
29. Jumper, J. et al. Highly accurate protein structure prediction with AlphaFold. *Nature* **596**, 583-589 (2021).
30. Rodrigues, C.H.M., Pires, D.E.V. & Ascher, D.B. DynaMut2: Assessing changes in stability and flexibility upon single and multiple point missense mutations. *Protein Sci.* **30**, 60-69 (2021).
31. Purvis, A. & Hohenester, E. Laminin network formation studied by reconstitution of ternary nodes in solution. *J. Biol. Chem.* **287**, 44270-7 (2012).
